# Human microbiome signatures of differential colorectal cancer drug metabolism

**DOI:** 10.1038/s41522-017-0034-1

**Published:** 2017-11-01

**Authors:** Leah Guthrie, Sanchit Gupta, Johanna Daily, Libusha Kelly

**Affiliations:** 1 Department of Systems and Computational Biology, Albert Einstein College of Medicine, Bronx, NY 10461 USA; 2 Department of Microbiology and Immunology, Albert Einstein College of Medicine, Bronx, NY 10461 USA

## Abstract

It is well appreciated that microbial metabolism of drugs can influence treatment efficacy. Microbial β-glucuronidases in the gut can reactivate the excreted, inactive metabolite of irinotecan, a first-line chemotherapeutic for metastatic colorectal cancer. Reactivation causes adverse drug responses, including severe diarrhea. However, a direct connection between irinotecan metabolism and the composition of an individual’s gut microbiota has not previously been made. Here, we report quantitative evidence of inter-individual variability in microbiome metabolism of the inactive metabolite of irinotecan to its active form. We identify a high turnover microbiota metabotype with potentially elevated risk for irinotecan-dependent adverse drug responses. We link the high turnover metabotype to unreported microbial β-glucuronidases; inhibiting these enzymes may decrease irinotecan-dependent adverse drug responses in targeted subsets of patients. In total, this study reveals metagenomic mining of the microbiome, combined with metabolomics, as a non-invasive approach to develop biomarkers for colorectal cancer treatment outcomes.

## Bowel cancer: human gut microbes can metabolize drugs

Differences in the microbial populations in the gut may help predict the likelihood of adverse reactions to a drug used to treat bowel cancer. Libusha Kelly, Leah Guthrie, and colleagues at Albert Einstein College of Medicine in New York examined the undesirable reactivation of the chemotherapy drug irinotecan by microbial enzymes in the gut. They identified an association between specific forms of microbial metabolic activity and drug metabolism. Sampling the microbial population of a patient’s gut may therefore offer a relatively non-invasive way to identify biomarkers predicting the likelihood of adverse reactions due to microbial metabolism. The research also suggests that using drugs to inhibit the activity of specific microbial enzymes in the gut might improve the outcome of some treatments. Modifying the microbial population prior to treatment may be another option.

## Introduction

The microbiome shapes the metabolic^1^ and immunological^2^ landscape of individuals in health and disease. Its plasticity can be leveraged for therapeutic interventions^3^ and to improve therapeutic outcomes.^4,5^ Recent studies have implicated gut microbiome metabolism at the gene^5^ and species^6^ level in driving the variability in patient drug response and toxicity. Thus, understanding the mechanisms of microbial mediated drug biotransformation and quantifying the microbial origins of variability in drug response may improve patient treatment outcomes.

One of few therapeutic drugs for which we have a mechanistic understanding of how the gut microbiome specifically influences drug metabolism is the colorectal cancer chemotherapeutic and prodrug irinotecan (CPT-11). CPT-11, in combination with fluorouracil and leucovorin, is one of three first-line treatments for metastatic colorectal cancer (CRC).^7,8^ CPT-11 is administered to patients intravenously and converted to its active form (SN-38) by carboxylesterases in the liver.^5,9^ It is inactivated by UDP-glucuronosyltransferases to a glucuronidated form (SN-38G) that enters the intestine via biliary excretion.^10,11^ Damage to intestinal epithelial cells and severe diarrhea can occur when SN-38G is reactivated by microbial β-glucuronidases (BGs) in the gut, which recognize the glucuronidated drug as a carbon source^5^. Adverse drug responses (ADRs) to CPT-11 vary substantially in patient populations,^10,11^ potentially reflecting inter-individual variation in gut metabolism of the excreted drug. When CPT-11 is administered as a single agent, 30–40% of patients experience grade 3–4 diarrhea,^12^ considered life-threatening and requiring hospitalization.^13^ More commonly, CPT-11 is administered as a part of treatment regimens including other therapeutics; here 11–37% of patients experience grade 3–5 diarrhea.^14^

Historically, oral antibiotics were used to reduce CPT-11 induced toxicity,^15^ however indiscriminant depletion of gut microbes may impair protective functions, including the ability to resist infection and the capacity to metabolize dietary substrates. Furthermore, gut microbiota depletion directly impacts chemotherapy treatment through a variety of mechanisms, including the prevention of beneficial crosstalk with the immune system.^16^ Recent efforts to reduce CPT-11 toxicity include targeted inhibition of microbial enzymes that convert the inactive form of the drug to its active form. Wallace et al., 2010, identified potent *Escherichia coli* BG inhibitors which substantially reduce CPT-11 induced toxicity in mice while having no effect on the orthologous mammalian enzyme.^5^

We hypothesized that patterns of BG gene abundance, and potentially other genes present in the gut microbiome, are linked to drug metabolism phenotypes and therefore may predict individual patient responses to drugs. Here, we have identified gut microbiome-derived metagenomic signatures linked to an individual’s microbial community level capacity to convert the inactive form of CPT-11, SN-38G, to the active form, SN-38, using high throughput genomics in combination with metabolomics to quantitate gut microbiota-produced metabolites of SN-38G.

## Results

### Subject characteristics

Fecal samples were collected from 20 healthy individuals; details of the cohort are provided in Table S1. The participants were healthy young adults without antibiotic exposure within 6 months prior to study enrollment.

### Human fecal microbiota mediated SN-38G metabolizer phenotypes

**Fig. 1:**
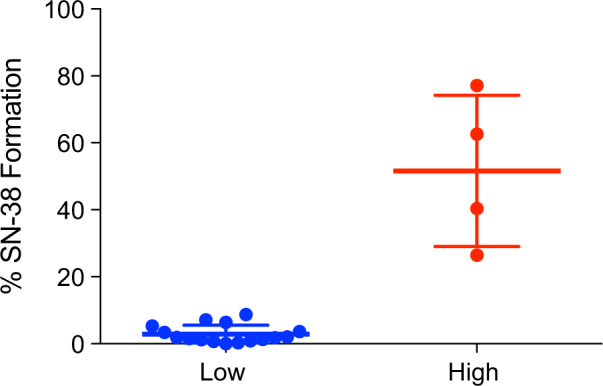
Two distinct metabolizer phenotypes or ‘metabotypes’ based on % SN-38 formation during a time course incubation of SN-38G with fecal samples from 20 individuals quantified by LC-MS/MS. Participants were sub-grouped into low (*n* = 16) and high (*n* = 4) metabolizer phenotypes. All samples were run in triplicate and values are the mean ± sem

We studied 20 microbiomes to characterize the variability in human gut microbiota mediated conversion of SN-38G into SN-38 and to determine the microbial basis of this variability. We first defined metabolism of SN-38G for each individual using time course ex vivo incubations of fecal extracts with SN-38G and targeted LC-MS/MS for the quantitation of SN-38 formed. We identified two distinct metabolizer phenotypes, or ‘metabotypes’, based on % SN-38 formation, which can be sub-grouped into low (0.04–8.72%) and high (26.46–77.11%) metabolizer phenotypes (Fig. 1).

### Specificity of assays to quantify SN-38G metabolizer phenotypes

Fecal BG activity is commonly measured based on the turnover of p-Nitrophenyl β-D-glucuronide (PNPG) to p-Nitrophenyl (PNP) using a plate-based absorbance assay.^17^ PNPG assays have the advantages of being faster, cheaper and more amenable to high throughput analysis. We therefore sought to determine whether the low and high metabotypes could be resolved by incubating fecal samples with PNPG and using a plate-based assay for quantification. We found no correlation between the targeted metabolomics assay and the PNPG assay (*R* = 0.2, *p* = 0.39) (Figure S1).

### Microbiome structure and composition across metabotypes

To determine if metabotypes were correlated with specific bacteria we profiled the taxonomic composition across metagenomes. At the phylum level, all samples were dominated by Bacteroidetes (mean relative abundance 64%) and Firmicutes (mean relative abundance 29%) (Figure S2a). At the family level samples vary widely independent of metabotype with a few dominant families. Bacteroidaceae were the most prevalent across most samples, making up to 77.59% of the family level relative abundance count within a participant (Figure S2b). Minor dominant taxa included Lachnospiraceae (mean relative abundance 22%) and Ruminococcaceae (mean relative abundance 9%). Profiling microbial community composition at multiple levels of resolution did not predict low or high metabotype via correlation analyses.

### Relationship between Enterobacteriaceae family and BG gene abundances

To determine whether Enterobacteriaceae family members, which have been the focus of efforts to inhibit BGs in the context of CPT-11 toxicity^5^ and other glucuronidated drugs,^18^ were more dominant in high metabolizers, and thus whether Enterobacteriaceae family distribution related directly to the abundance of Enterobacteriaceae BGs, we first used STAMP^19^ to assess differential abundance. We found that samples within the high metabotype group did not have an increased abundance of Enterobacteriaceae members; instead, this family was variably distributed across samples (Figure S2b).

**Fig. 2:**
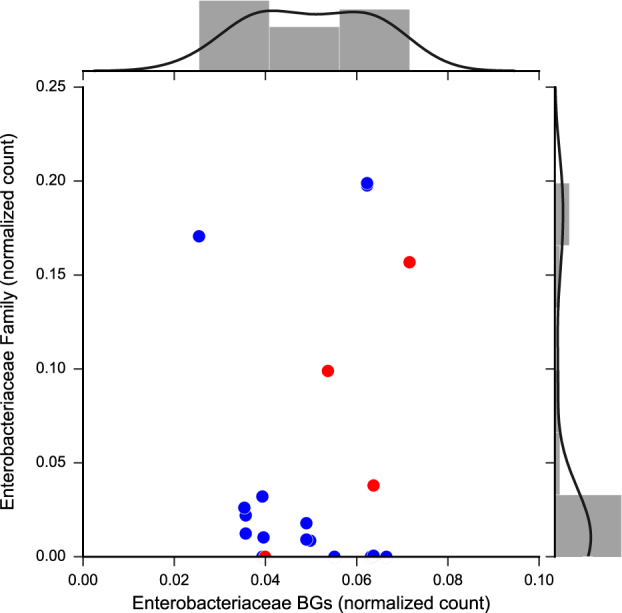
Relationship between the distribution of the Enterobacteriaceae family and Enterobacteriaceae β-glucuronidase. Enterobacteriaceae BGs were identified from the RefSeq, UniProtKB, and PDB protein databases, clustered at 99% identity, and mapped to assembled metagenomes

To look specifically at Enterobacteriaceae-like BGs in our samples we assessed their evenness using the Simpsons Equitability (ED) metric^20^ and distribution (Fig. 2). Enterobacteriaceae BGs are more evenly shared (ED = 0.938) across the samples while the Enterobacteriaceae family members are more variably distributed (ED = 0.629) across samples independent of metabotypes.

### Diversity, abundance, and mobility of BGs across metabotypes

**Fig. 3:**
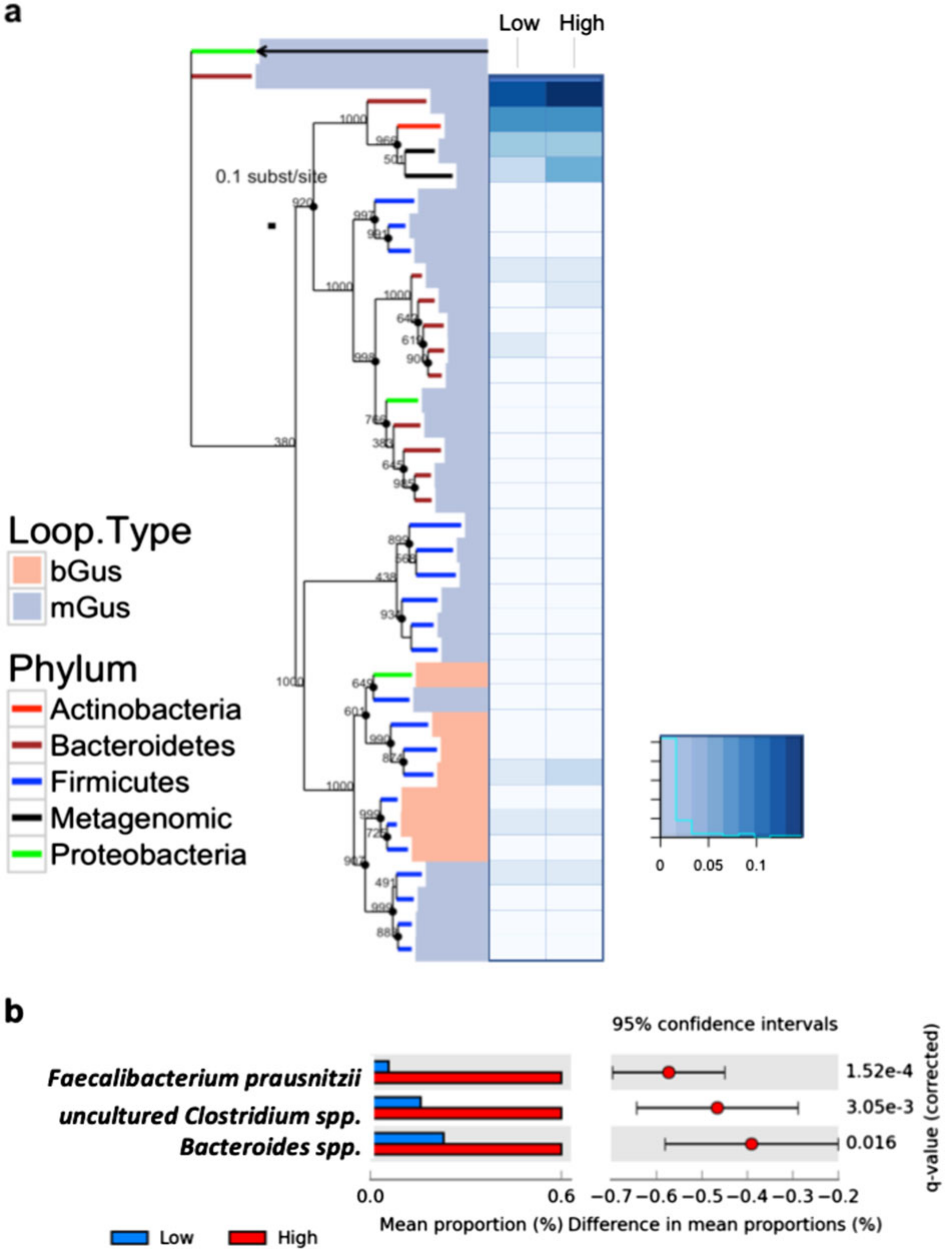
Phylogenetic distribution and abundance of loop positive and negative BGs of healthy individuals. **a** The BG tree is rooted using the *E. coli* and *B. thetaiotamicron* β-galactosidase sequences, the *E. coli* sequence is indicated by an arrow. Phylum-level taxonomy is indicated by branch color. Salmon colored bars represent loop positive sequences while the light blue bars indicate loop negative sequences as defined by Wallace, 2015. The adjoining heat map displays the relative abundance of BG sequences represented in the tree with values normalized on a scale from 0, being least abundant to 1, being most abundant. **b** Differentially abundant BGs between the low and high metabotype individuals were determined based on the Welch’s *t*-test, two-sided with a Storey FDR, adjusted *q*-value < 0.05 and followed by an effect size filter (ratio of proportions effect size < 2.00)

We observed that BG gene abundance is variable among healthy subgroups of people (Fig. 3a). To determine whether particular sets of BG predict the low or high metabotypes, we first compiled an extended database of BG sequences, including BG sequences from the healthy individuals in the Human Microbiome Project. We next mapped participant metagenomes to this database. We identified BGs from a predicted uncultured *Clostridium spp., Faecalibacterium prausnitzii* and a *Bacteroides* species that were significantly differentially abundant between high and low metabolizers (Fig. 3b).

### BGs and the progression to colorectal cancer

BG activity is greater in colorectal cancer patients^21^ and we therefore asked whether the BGs present in the healthy participant sample set reflected the BGs present in carcinoma patients. Using the ref. 22 dataset of patients (*n* = 156) with advanced adenomas, carcinomas and age matched healthy controls, we found that an overlapping set of BGs are carried by the high metabotype and carcinoma patients (Figure S3).

### A putative transport mechanism for SN-38G into bacterial cells

**Fig. 4:**
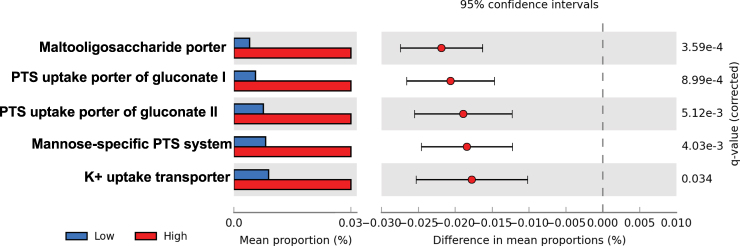
Functional profiling of the fecal microbiome transporter diversity using metagenomics. Metagenomic reads were mapped to the Transporter Classification Database (TCDB). Differential abundance between metabotypes was determined using the Welch’s *t*-test, two-sided with a Storey FDR, adjusted *q*-value < 0.05 and followed by an effect size filter (ratio of proportions effect size < 2.00)

Bacterial metabolism of SN-38G requires entry into the bacterial cell, however the specific genes involved in this process are unknown. To determine whether there were shifts in the sets of transporters present in low and high metabotypes, assembled metagenomes were mapped against the Transporter Classification Database.^23^ We identified transporters involved in carbohydrate uptake that were more abundant in the high metabotypes (Fig. 4). Several transporter proteins from the Carbohydrate Uptake Transporter-1 (CUT1) family were differentially abundant and enriched in the high metabotype samples (Enzyme Commission ID: 3.A.1.1). Other proteins that were more abundant in the high metabotype were part of the Gluconate:H+ Symporter (GntP) Family (2.A.8), the PTS Mannose-Fructose-Sorbose (Man) Family (4.A.6), the Heavy Metal Efflux (HME) Family (2.A.6.1), and the Holin LLH (Holin LLH) Family (1.E.26). These results link metabotypes to sets of transporters at the level of metagenome; the specific genomes of origin were not resolved.

### Community-level microbiome changes in metabolism across metabotypes

**Fig. 5:**
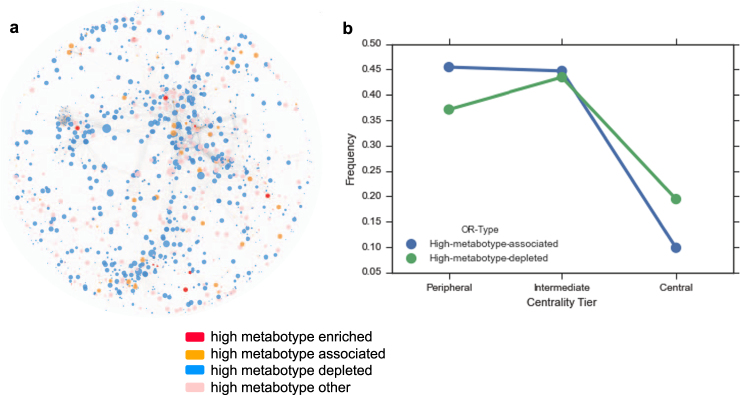
Metabotype associated enzymes in the context of community level metabolic network features. **a** Community level metabolic network with nodes representing KEGG orthologous groups and colored by odds ratio type. **b** Frequency of associated (depleted, associated and enriched) and non-associated enzymes across the central, intermediate and peripheral centrality tiers

To determine whether there are differences in microbial metabolism at the broader level of the entire metagenome between metabotypes we used topological analyses of an enzyme-centric metabolic network. Previous studies on bacterial metabolic network dynamics and structure found that network topological features relate to an enzyme’s relational position in a pathway (e.g., first step, intermediate step, nutrient uptake step) and that peripheral enzymes have higher rates of horizontal gene transfer.^24–27^ To examine variation in gene abundance in the context of community level metabolic network characteristics we identified enriched genes across the low and high metabotypes using the odds ratio and differential abundance score as defined by ref. 27 High metabotype-enriched genes (OR > 2), are predominantly involved in carbohydrate and amino acid metabolism (Fig. 5a) (Table S2).

To identify central vs. peripheral enzymes involved in metabolic pathways, a network was constructed based on the Kyoto Encyclopedia of Genes and Genomes (KEGG) orthologous groups present in the metagenomes and betweenness centrality was calculated using Cytoscape.^28,29^ To determine whether the distribution of centrality scores differed between high metabotype-associated vs. non-associated KEGG groups we used a Wilcoxon rank-sum test with a *p* value < 0.05 cutoff for significance. High-metabotype enzymes have lower centrality (Fig. 5b) and are more peripheral in the community metabolic network, suggesting that the high metabotype can be distinguished from the low by a greater abundance of enzymes involved in processes that potentially interface with the gut environment.

## Discussion

**Fig. 6:**
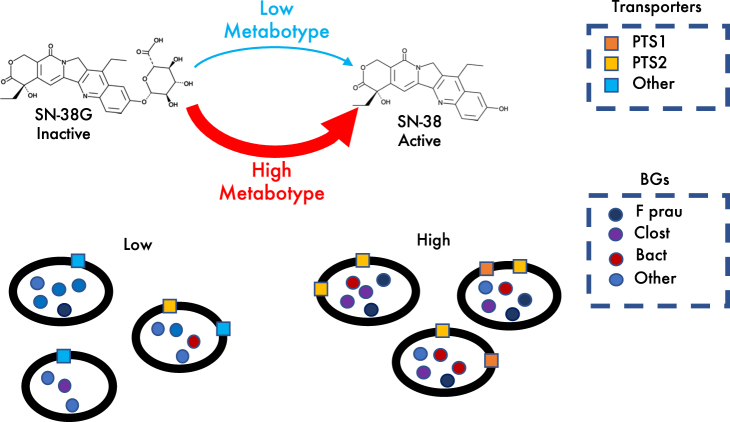
Proposed model of low and high metabotype human gut microbiome interactions with SN-38G. SN-38G enters cells via carbohydrate transporters that are more prevalent in high metabotype individuals. Following cell entry, high metabotypes metabolize SN-38G more efficiently using BGs from a predicted uncultured *Clostridium spp., Faecalibacterium prausnitzii* and a *Bacteroides* species

Glucuronidation is a common modification of xenobiotics as part of phase II drug metabolism. Both sick and healthy individuals ingest a variety of glucuronidated compounds—prescribed or purchased over the counter—that are excreted via the biliary route.^30,31^ In addition to irinotecan, examples include paracetamol, codeine, chloramphenicol, vitamins, and tamoxifen.^30–34^ An unanswered question is how the microbiome influences variability in community level conversion efficiency of these glucuronidated substrates. Our study presents a framework to address this question. We quantify the microbial basis of variability in SN-38G turnover, a key determinant of irinotecan-induced toxicity. We find associations between the efficient microbiota-mediated turnover of SN-38G and specific microbial BGs from abundant gut species and we propose a putative transport mechanism for SN-38G entry into bacterial cells (Fig. 6).

Using LC-MS/MS for the quantitation of gut microbiota produced metabolites of SN-38G, we stratified our patients into low (0.04–8.72% hydrolysis; *n* = 16) or high (26.46–77.11% hydrolysis; *n* = 4) metabolizer phenotypes or 'metabotypes' (Fig. 1). Notably, the metabotypes identified via LC-MS/MS would be indistinguishable in the PNPG-based assay of BG activity, suggesting substantial diversity in glucuronide substrate utilization among BG enzymes (Figure S1). Most samples from our healthy donors had a low microbiota mediated biotransformation of SN-38G, which is consistent with the clinical prevalence of ADRs.^12,14,35^ Accordingly, our data provide the first quantitative experimental evidence that inter-individual variation in total community level microbiome bacterial protein activity can result in differential metabolism of SN-38G. Future work is necessary to determine whether fecal turnover correlates with irinotecan-induced toxicity and whether the microbiome might therefore serve as an accurate predictor of patient ADR risk.

We identified three BGs that were more abundant in the high metabolizers and have not been previously associated with deconjugation of therapeutics (Fig. 3b). All were identified based on sequence homology to known or predicted glycosyl hydrolyases or BGs from genera that have experimentally confirmed BG activity. One of the identified BG was most similar to a predicted uncultured *Clostridium* species. Among *Clostridium* species, *C. perfringens* has been found to have higher BG activity than *E. coli*, *Staphylococcus*, *Corynebacterium spp*., *Bacillus spp*., *Enterococcus spp*., *Acinetobacter spp*., *Streptococcus spp*., and *Klebsiella spp*.^36^ However, many other *Clostridium spp*. have been reported to have little to no BG activity^37^ suggesting gene loss and a polyphyletic relationship among some species for BGs, as observed in other carbohydrate metabolizing genes in other bacterial species.^38^ The second BG identified was most similar to a *F. prausnitzii* glycosyl hydrolyase. Notably *F. prausnitzii* subspecies vary in the presence or absence of BG activity.^37,39^ This is suggestive of selection on sets of BG genes in which the host diet potentially drives the prevalence of BG activity among *Faecalibacterium.* ^39,40^ Redirection of carbohydrate metabolism capacity has been demonstrated experimentally in *Bacteroides thetaiotaomicron*, which diverts its carbohydrate metabolism capacity from dietary to host polysaccharides, such as the mucus layer overlying the epithelium, in a nutrient availability-dependent manner.^40^ Low carbohydrate diets may promote species level adaptive foraging of carbohydrates from xenobiotics and host sources. The third BG is homologous to a *Bacteroides* species glycosyl hydrolase. BG activity is broadly found in *Bacteroides.* ^37^ It is notable that all three of these high metabotype-associated BGs are phylogenetically distinct and have different structural features from the well-studied *E. coli* G (Fig. 3a),^4^ suggesting that pairing biochemical assessment of phylogenetically diverse BGs with genomically defined metabotypes will be an important step towards designing better inhibitors to target microbial BGs.^4^ Our findings suggest that a diverse set of BGs may need to be targeted for inhibition to be successful in the context of the human gut.

In addition to species level diversity in BG activity we also considered whether differences in substrate uptake preference played a role in distinguishing the high and low metabotypes. To date, no mechanism of entry for SN-38G into bacterial cells has been established. We identified two Gluconate:H+ Symporter (GntP) Family (2.A.8) transporters that were more abundant in the high metabotype group (Fig. 4). These transporters are involved in a transport system that was first characterized in *Streptococcus pneumoniae*, which is dependent on carbohydrates for growth while living in the low carbohydrate environment of the human airway.^41^ GntP family transporters are a part of the gluconate transport system, which releases carbohydrates from glycol-conjugates that are N or O linked. This system also involves a phosphoenolpyruvate-dependent phosphotransferase system (PTS), an unsaturated glucuronyl hydrolase (Ugl) and a hyaluronate lyase (Hyl) capable of cleaving glycosaminoglycan hyaluronic acid for use as a carbon source.^41^ The abundance of the PTS system sugar specific EII component in high metabotype samples represents a potential mechanism, heretofore unknown, by which the glucuronidated drug enters the bacterial cells efficiently.

The results here highlight close associations between BG activity, phylogenetic diversity, and clinically important metabolites and provide insight into the broad variability in BG abundance and activity in healthy individuals. Notably, BG activity is greater in colorectal cancer (CRC) patients and with meat consumption; it is hypothesized that BG mediated deconjugation of heterocyclic amine produces reactive metabolites that damage colonic mucosal cells,^21^ suggesting that BGs may play a role in both the etiology and treatment efficacy for CRC. Colorectal cancer can be characterized by alterations in the microbiome^42,43^ and consequentially colorectal cancer patients may have non-overlapping sets of BGs with our healthy EMP cohort. We found that high metabotypes and carcinoma patients carry overlapping sets of BGs, however more work is needed to assess if BG metabolism in colorectal cancer patients correlates with the same microbiome markers identified here in healthy subjects.

Consistent with previous studies profiling the gut microbial community of healthy individuals we found that taxa are relatively stable across the healthy fecal microbiomes at the phylum level and are variable at the family to species levels.^44^ Taxonomic variation among healthy individuals favors the hypothesis that the healthy human microbiome may be instead defined by core functional capabilities.^45^ These findings lead us to hypothesize that taxonomic diversity and functional traits such as the SN-38G metabotypes might not be linked. For example, the non-steroidal anti-inflammatory drug, diclofenac, like SN-38, is detoxified via glucuronidation and excreted into the gut and reactivated in a subset of patients resulting in enteropathy.^18^ In mice, co-administration of diclofenac with the fluoroquinolone antimicrobial agent, ciprofloxacin, reduces diclofenac-induced toxicity.^18^ The authors implicate microbial BGs and hypothesize that Enterobacteriaceae BGs are the major players in both diclofenac-glucuronide and SN-38G metabolism and the resulting adverse responses.^18^ Furthermore, due to the effectiveness of inhibitors designed against the *E. coli* BG to reduce irinotecan toxicity in mice,^5^ we looked at the relationship between the Enterobacteriaceae family and Enterobacteriaceae-like BGs across samples and found that Enterobacteriaceae-like BGs are widely distributed across samples and incongruent with taxonomic abundance (Fig. 2). These results suggest that the Enterobacteriaceae BG can be more prevalent in individual microbiomes than Enterobacteriaceae family members, suggesting that this gene is likely horizontally exchanged in microbial populations and that 16S-based taxonomic profiling would not be sufficient to predict Enterobacteriaceae BG abundance.

In bacteria, metabolic network topology is a product of the functional interdependence between genes such that central genes in a network are proposed to be involved in intermediate steps of metabolism while peripheral genes are involved in either the first (e.g., nutrient uptake) or last steps (e.g., a product that interacts with the gut environment).^26,46^ Differences in the gain and loss of peripheral enzymes may explain the enzyme-level variability associated with high and low metabotypes. Peripheral enzymes in our samples span multiple functional categories but are dominated by carbohydrate metabolizing enzymes (Table S2), suggesting that differences in the utilization of available carbohydrates may be a distinguishing feature between metabotypes.

What are additional mechanisms driving the gut microbiome signatures that are associated with our metabotypes? This study suggests that a variable and diverse set of BGs may be critical to the metabolic efficiency of SN-38G transformation. Additional work to more thoroughly characterize these BGs is necessary, for example, examining the timescales of BG expression post SN-38G exposure. To fully understand the scope and scale of metabolic diversity, additional work quantifying the efficiency of SN-38G turnover in more individuals will reveal how generalizable our metabotypes are to larger populations. Future work will address correlations between BG activity and drug response in patients receiving regimens containing CPT-11. Our high metabotype-associated BGs are promising targets for predicting and modulating adverse drug responses in patients. Metagenomic assessment of carbohydrate-active enzymes represents a non-invasive approach to developing biomarkers for colorectal cancer treatment outcomes and is a first step towards engineering microbial community composition to promote human health.

## Materials and methods

### Participant recruitment and sample collection

To examine the association between SN-38G metabolites and the microbiome 20 healthy individuals were recruited to participate in the Einstein Microbiome Project (EMP) for one-time fecal sampling. No previous study has investigated the reactivation of SN-38G by the gut microbiota in a comparable manner; therefore, no power analysis for the sample size could be performed. The study was approved by the Albert Einstein College of Medicine Institutional Review Board. Subjects were recruited via flyers posted at Albert Einstein College of Medicine. Subjects who were >18 years of age, had no health conditions and had not used antibiotics within the prior 6 months were enrolled and provided informed consent. Age, gender and BMI were recorded. Participants collected fecal samples using the Commode specimen collection system (Fisher) and delivered them to the laboratory within 2 hours of being produced. Samples were immediately stored at −80 °C. After thawing, samples were divided into 0.3 g aliquots for DNA extraction and metabolic analysis.

### Time course ex vivo incubations of fecal samples with SN-38G

To quantify the microbiome metabolism of SN-38G we carried out ex vivo incubation of SN-38G with each fecal sample as follows: To remove debris, 0.3 mg of each fecal sample was mixed with 3 ml of Dulbecco’s phosphate-buffered saline, homogenized, centrifuged at 10,000×*g* for 15 min at 4 °C and the supernatant was collected for further processing. A final concentration of 200 ug/ml total protein per sample was prepared using the Bradford assay. Each sample was then incubated with 100 uM SN-38G at 37 °C. Reactions were terminated at 0, 1.5, and 3 min by removing a sample aliquot and adding a quenching solution containing the internal standard, 100 uM hydroxycampotothecin-d5 (ISTD), in 50% methanol. The ISTD is a compound similar in structure to SN-38 that is not metabolized by the gut microbiota. Samples were centrifuged at 12,000×*g* for 10 min and 5 ul of supernatant was added to 45 ul of 10% methanol.

### LC-MS/MS analysis

The concentrations of SN-38G, SN-38 and the ISTD in the fecal extracts were determined by multiple reaction monitoring, focusing on selective ions for SN-38 (393.2→349), SN-38G (579.0 →394.1), and ISTD (371.1→327.1) (Table S3). The instrument used, the Agilent G6490 Triple Quadrupole Mass Spectrometer, was operated in the positive ionization mode and connected online to a 1290 Infinity series UHPLC. Mobile phase A was aqueous with 10% acetonitrile and 0.1% formic acid to maintain the lactone form of SN-38. Mobile phase B was composed of 100% acetonitrile. Each sample was run in triplicate at a flow rate of 0.350 ml/min with blanks consisting of sample buffer placed between each set of samples and the variance in triplicate points was determined (Table S4). A calibration curve was established for each metabolite and the ISTD in both methanol and using a pooled fecal extract to determine the lower limit of detection and lower limit of quantitation. This work was carried out with the Albert Einstein College of Medicine Proteomics Core.

### DNA extraction, library construction, and shotgun metagenomic sequencing

The PowerFecal DNA isolation kit (MO BIO Laboratories, Inc., San Diego, CA) was used to extract DNA per protocols established by the Human Microbiome Project.^47^ Library construction and sequencing was carried out at the New York Genome center using the TruSeq Nano DNA LT Library Preparation Kit (Illumina, Inc., San Diego, CA), which generates 450 bp libraries and sequenced on the HiSeq 2500 System at 2 × 125 bp read length resulting in ~250 M reads.

### Read filtering, assembly and gene calling

The clc_quality_trim program was used to quality filter paired reads by removing bases on both ends with quality scores below 20 and with an allowance of 10% low-quality bases. Filtered reads were assembled with the SPADES assembler (v3.7.1)^48^ with default parameters. Gene calls were made using Prodigal^49^ with the –p meta flag and otherwise default parameters.

### Shotgun metagenomic sequence analysis

The assembled metagenomes were mapped against the following functional databases using USEARCH version 8 with an e-value cutoff of 1e-40 in order to ensure longer sequence hits for improved taxonomic and functional resolution: KEGG EC/KO (*n* = 2,000,708),^50^ Transporters (*n* = 5,7229),^23^ Carbohydrate active enzyme (CAZy) (*n* = 7215),^51^ and a curated database of both cultured and metagenomically identified BGs developed in-house.^52^ The abundance of KEGG orthologous groups and modules were determined using the HUMAnN pipeline^53^ with default parameters. For the KEGG, Transporters and CAZy databases abundance counts were aggregated across samples. Differential abundance of pathways and genes between low and high metabotypes was performed using STAMP’s implementation of the Welch’s *t*-test^19^ with a Storey FDR adjusted *q*-value < 0.05 and followed by an effect size filter (ratio of proportions effect size < 2.00). For all analyses carried out in STAMP we used the Welch’s *t*-test due to the unequal size of the metabotypes groups and the approximate normality of the BG and transporter data. The Shapiro–Wilk normality test implemented in R (R version 3.3.1)^54^ demonstrated no significant departure from normality based on the distribution of hits in the the BGs (*p* = 0.863) and Transporters (*p* = 0.880) databases.

### Microbial taxonomic abundance estimates

For taxonomic profiling the reads were mapped against the MetaPhlAn marker gene database^55^ using Bowtie 2.2.1^56^ with default parameters. MetaPhlAn scripts were used to extract normalized abundance counts across phyla with default parameters.^55^ Statistical differences between the stratified metabolizer phenotypes, low and high, were determined using STAMP’s implementation of the Welch’s *t*-test^19^ with a Storey FDR, adjusted *q*-value < 0.05 and followed an effect size filter (ratio of proportions effect size < 2.00).

### Characterizing phylogenetic diversity of BGs across samples

To characterize the phylogenetic diversity and distribution of BGs in both our collected patient samples (EMP) as well as the metagenomes from patients with colorectal cancer from the Feng et al. study,^22^ we included BG sequences identified as a part of the Human Microbiome Project extracted from the IMG-M database^57^ by searching for all assembled scaffolds in each patient sample for genes annotated as EC:3.2.1.31, the Enzyme Commission^58^ identifier for BGs. USEARCH 8 was used to search the metagenomes against our protein database with an e-value cutoff of e-40 and a bit score of 200. Patient metagenomes were also mapped to a database of Enterobacteriaceae BGs identified from the RefSeq, UniProtKB and PDB protein databases, clustered at 99% identity. To examine the distribution of BGs in a colorectal cancer patient cohort, gene calls from the ref. 22 shotgun metagenomic study of patients with advanced adenomas, carcinomas and age matched controls, were compared to this database of cultured and metagenomically identified BGs. This study included in-depth nutritional survey of participants, extensive clinical data collection and single time-point shotgun metagenomic profiling.

A community level phylogenetic tree was constructed by aligning the in-house database of cultured and metagenomically identified BGs using MUSCLE^59^ and PhyML^60^ with 1000 bootstrap replicates, a JTT model of substitution, and otherwise default parameters. The *E. coli* and *B. thetaiotamicon* β-galactosidase sequences were used as out-groups, as described previously^4^.

### Enrichment analysis of KEGG orthologous groups

To determine if there were differences in KEGG orthologous groups (KOs) associated with the variation in SN-38G metabolism we identified KOs associated with each metabolism phenotype (‘metabotype’). An odds ratio was calculated for each enzyme as described by Greenblum et al.^27^ The differential abundance score was defined as the absolute value of the fold change in odds ratio (OR), abs[log2(OR)]. Differential abundance scores were classified as high metabotype-enriched (OR > 2), high metabotype associated (OR > 1), high metabotype-depleted (OR < 0.5) and high-metabotype-other (OR > 0.5 and <1) as described previously.^27^ To identify KOs that are associated with the high metabotype, the abundance of each enzyme in the set of samples obtained from high metabotype individuals was compared with its abundance in low metabotype individuals.

### Community-level metabolic network construction

To determine whether high metabotype-enriched enzymes occupy a similar role in the context of a metagenome wide community level metabolism, a community-level metabolic network was constructed from the KOs present across all samples. Nodes in the network represent the enzymes of KOs and directed edges between nodes indicate that a product of the first enzyme is a substrate of the second enzyme.^27^ Using a list of the KOs present in the samples as input, the substrates and products associated with each KO were identified using the MMNET R package^61^ and the resulting igraph file was exported and analyzed using Cytoscape.^28^

### Enzyme-centric metabolic network analysis

Betweeness centrality was calculated using the Network Analyzer tool in Cytoscape.^28^ A Wilcoxon rank-sum test was used to compare the distribution of topological features of metabotype-associated enzymes with the values obtained for non-associated enzymes with a *p* value < 0.05 cutoff for significance. The Spearman correlation test was used to examine the correlation between an enzyme’s differential abundance scores and each topological feature in the network with a *p* value < 0.05 cutoff for significance. All enzymes were binned into three centrality tiers, central, intermediate and peripheral as described in ref. 27 A hypergeometric enrichment test was used to examine the over-representation of host state-associated enzymes in each centrality tier with a *p* value < 0.05 cutoff for significance.

### Data availability statement

The high-throughput sequence data have been deposited in the National Center for Biotechnology Information (NCBI) BioProject database with project accession PRJNA373879. The publically available protein calls for the colorectal cancer metagenomes are available here. http://gigadb.org/dataset/100140. The authors declare that all other data supporting the findings of this study are available within the article and its Supplementary Information files, at https://github.com/kellylab/Microbiome_CRCdrug_Turnover or from the corresponding author on request.

## Electronic supplementary material

Supplemental Information
