# Supplementary material for: Human microbiome signatures of differential colorectal cancer drug metabolism
Source: NPJ Biofilms Microbiomes. 2017 Nov 1;3:27. doi: 10.1038/s41522-017-0034-1 (PMC5665930; doi:10.1038/s41522-017-0034-1)
Supplement: Supplementary file 1 — Supplemental Information [file 41522_2017_34_MOESM1_ESM.pdf]

Supplementary Information

Human microbiome signatures of differential colorectal cancer drug metabolism

Leah Guthrie[1], Sanchit Gupta[2], Johanna Daily[2], Libusha Kelly[1,2]
[1]Systems and Computational Biology; [2]Microbiology and Immunology at Albert Einstein College of Medicine

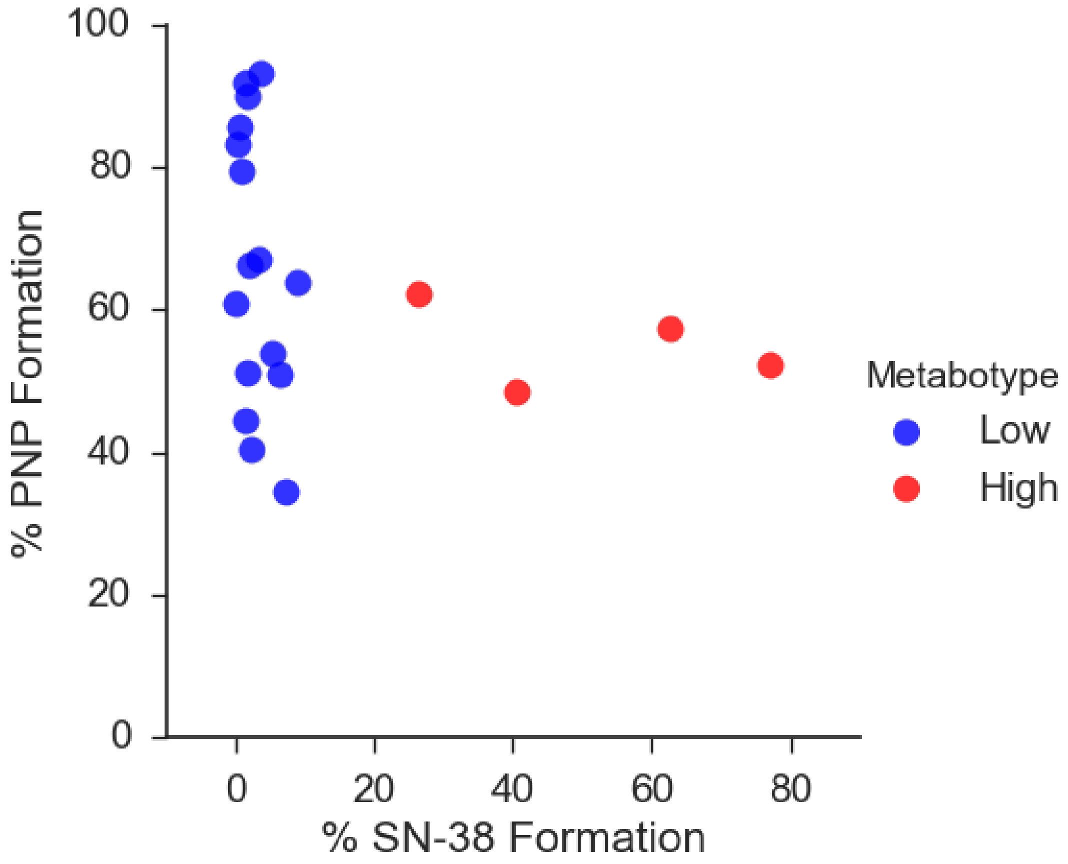

% PNP Formation
100
80
60
40
20
0
0
20
40
60
80
% SN-38 Formation
Metabotype
Low
High

(a)

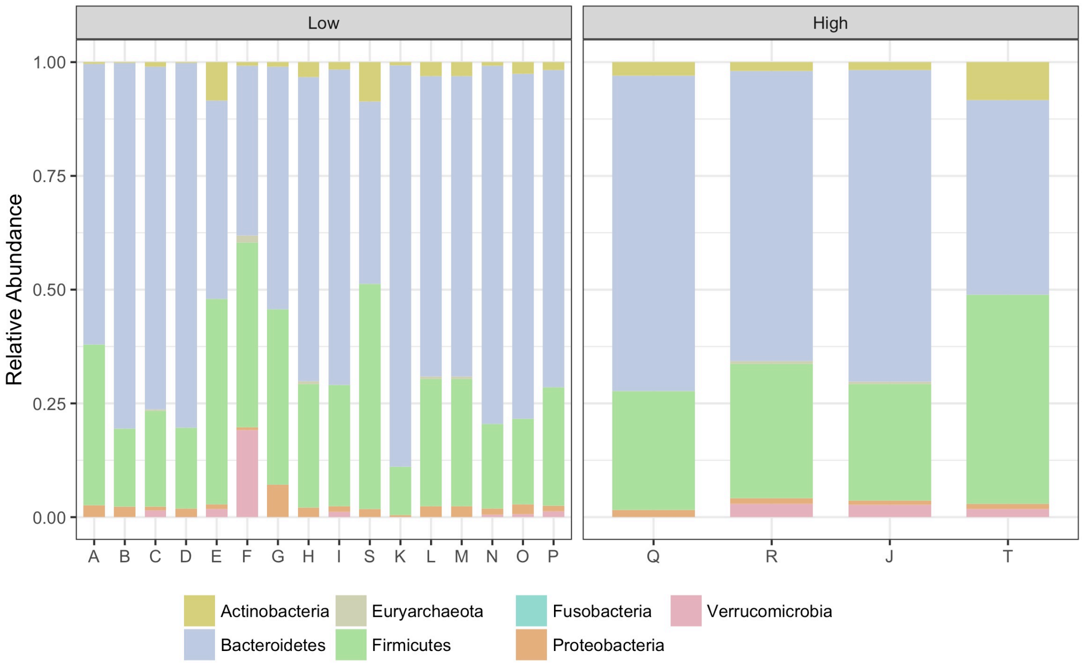

Low
High
Relative Abundance
1.00
0.75
0.50
0.25
0.00
A B C D E F G H I S K L M N O P
Q R J T
Actinobacteria
Euryarchaeota
Fusobacteria
Verrucomicrobia
Bacteroidetes
Firmicutes
Proteobacteria

(b)

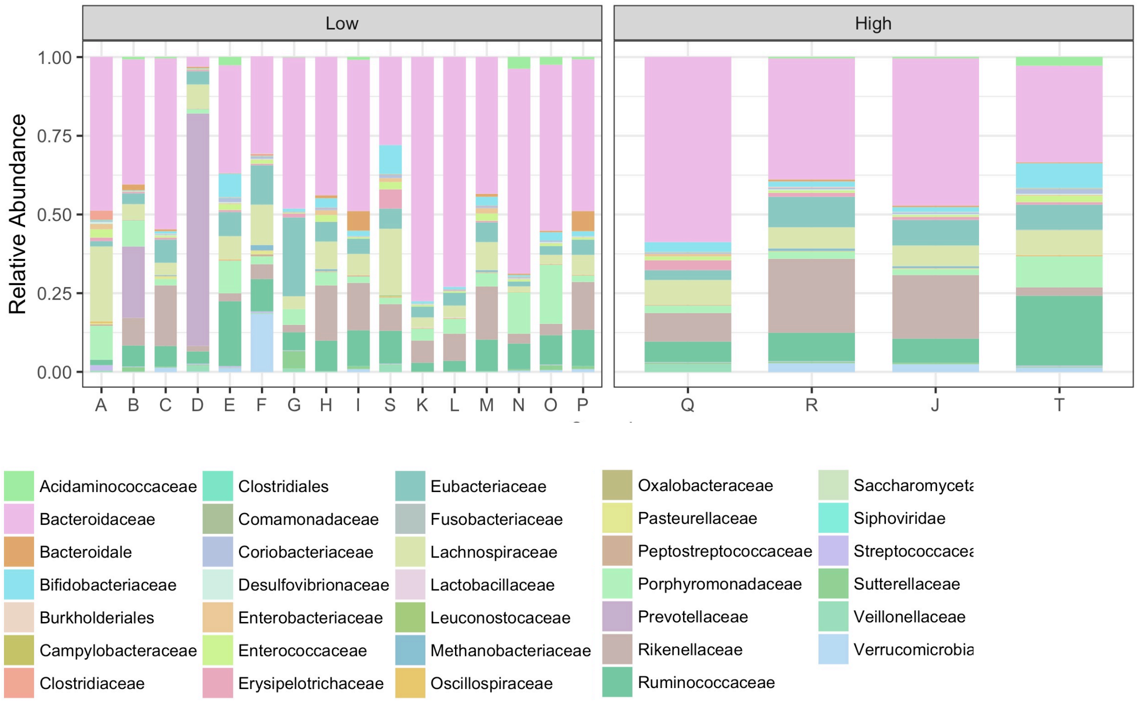

Low
High
Relative Abundance
1.00
0.75
0.50
0.25
0.00
A B C D E F G H I S K L M N O P
Q R J T
Acidaminococcaceae
Bacteroidaceae
Bacteroidale
Bifidobacteriaceae
Burkholderiales
Campylobacteraceae
Clostridiaceae
Clostridiales
Comamonadaceae
Coriobacteriaceae
Desulfovibrionaceae
Enterobacteriaceae
Enterococcaceae
Erysipelotrichaceae
Eubacteriaceae
Fusobacteriaceae
Lachnospiraceae
Lactobacillaceae
Leuconostocaceae
Methanobacteriaceae
Oscillospiraceae
Oxalobacteraceae
Pasteurellaceae
Peptostreptococcaceae
Porphyromonadaceae
Prevotellaceae
Rikenellaceae
Ruminococcaceae
Saccharomyceta
Siphoviridae
Streptococcacea
Sutterellaceae
Veillonellaceae
Verrucomicrobia

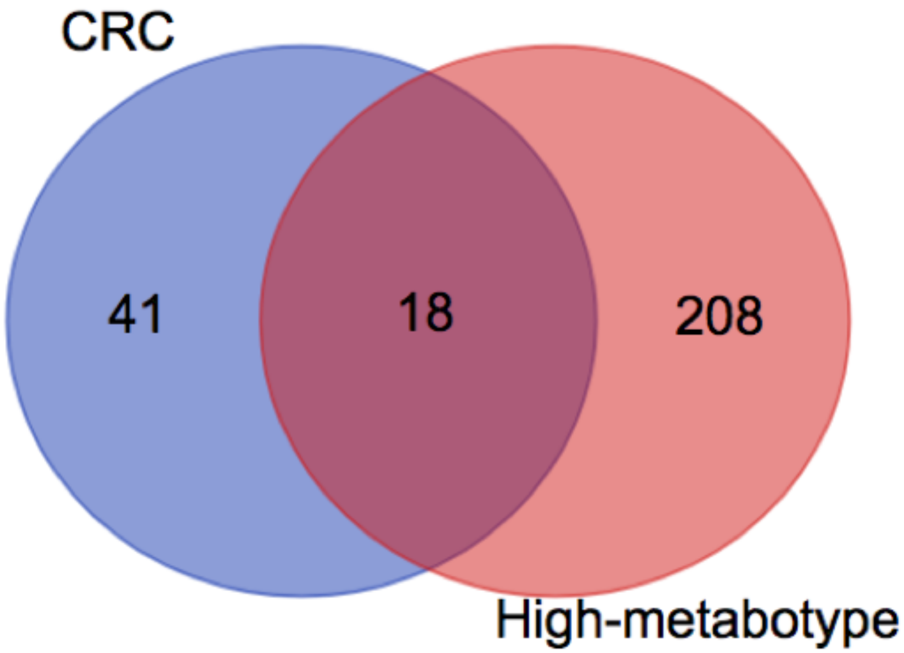
CRC
41
18
208
High-metabotype

Supplementary Figure Legends

Figure S1. No correlation between assays of BG activities of human fecal extracts using p-NPG and SN-38G as substrates. The 20 healthy fecal samples were processed as described for LC-MS/MS analysis. The conversion of p-Nitrophenyl β-D-glucuronide (PNPG) to p-Nitrophenyl (PNP) was determined using a plate-based absorbance assay as described by *Wallace et al, 2010*[1].

Figure S2. Microbial carriage is uniform at (a) the Phylum level, while variable at (b) the family level, but unlinked to metabotypes. Taxonomic abundance was determined using MetaPhlAn[2] and collapsed at the level of Phylum and family. Statistical differences between the stratified metabolizer phenotypes, low and high, were determined using STAMP's implementation of the Welch's t-test[3] with a Storey FDR, adjusted q-value < 0.05 and followed an effect size filter (ratio of proportions effect size < 2.00).

Figure S3. High metaboype participants and carcinoma patients carry overlapping sets of BGs. To examine the distribution of BGs in a colorectal cancer patient cohort, gene calls from the Feng et al, 2015[4] shotgun metagenomic study of patients with advanced adenomas, carcinomas and age matched controls, were compared to a database of cultured and metagenomically identified BGs. BGs carried by both high metabotype participants and carcinoma patients were identified and plotted in a Venn diagram.

Table S1. Participant characteristics

ID	AGE	SEX	BMI
A	25	Female	Normal
B	23	Female	Normal
C	24	Female	Normal
D	25	Male	Normal
E	27	Female	Normal
F	29	Female	Normal
G	29	Male	Overweight
H	21	Female	Normal
I	34	Male	Normal
J	28	Male	Normal
K	28	Female	Underweight
L	37	Male	Overweight
M	24	Male	Normal
N	29	Male	Normal
O	23	Male	Overweight
P	23	Female	Normal
Q	25	Female	Normal
R	36	Female	Normal
S	25	Female	Normal
T	28	Female	Normal

Table S2. Topological features of KEGG orthologous groups across all metagenomes

GID	OR-type	BetweennessCentrality	Centrality Tier
K14273	high-metabotype-depleted	0	peripheral
K01204	high-metabotype-depleted	0	peripheral
K01505	high-metabotype-depleted	0	peripheral
K09457	high-metabotype-depleted	0	peripheral
K02230	high-metabotype-depleted	0	peripheral
K00929	high-metabotype-depleted	0	peripheral
K03800	high-metabotype-depleted	0	peripheral
K00729	high-metabotype-depleted	0	peripheral
K01259	high-metabotype-depleted	0	peripheral
K02474	high-metabotype-depleted	0	peripheral
K00285	high-metabotype-depleted	0	peripheral
K02749	high-metabotype-depleted	0	peripheral
K03403	high-metabotype-depleted	0	peripheral
K01041	high-metabotype-depleted	0	peripheral
K03367	high-metabotype-depleted	0	peripheral
K01452	high-metabotype-depleted	0	peripheral
K10797	high-metabotype-depleted	0	peripheral
K03927	high-metabotype-depleted	0	peripheral
K01525	high-metabotype-depleted	0	peripheral

K01661	high-metabotype-depleted	0	peripheral
K00355	high-metabotype-depleted	0	peripheral
K00588	high-metabotype-depleted	0	peripheral
K00097	high-metabotype-depleted	0	peripheral
K01212	high-metabotype-depleted	0	peripheral
K14534	high-metabotype-depleted	0	peripheral
K00073	high-metabotype-depleted	0	peripheral
K02744	high-metabotype-depleted	0	peripheral
K06281	high-metabotype-depleted	0	peripheral
K01820	high-metabotype-depleted	0	peripheral
K06164	high-metabotype-depleted	0	peripheral
K01198	high-metabotype-depleted	0	peripheral
K00791	high-metabotype-depleted	0	peripheral
K12503	high-metabotype-depleted	0	peripheral
K01470	high-metabotype-depleted	0	peripheral
K03429	high-metabotype-depleted	0	peripheral
K00803	high-metabotype-depleted	0	peripheral
K01076	high-metabotype-depleted	0	peripheral
K02750	high-metabotype-depleted	0	peripheral
K00983	high-metabotype-depleted	0	peripheral
K01207	high-metabotype-depleted	0	peripheral
K06606	high-metabotype-depleted	0	peripheral

K00950	high-metabotype-depleted	0	peripheral
K01878	high-metabotype-depleted	0	peripheral
K03340	high-metabotype-depleted	0	peripheral
K00067	high-metabotype-depleted	0	peripheral
K01667	high-metabotype-depleted	0	peripheral
K09883	high-metabotype-depleted	0	peripheral
K10012	high-metabotype-depleted	0	peripheral
K01892	high-metabotype-depleted	0	peripheral
K02472	high-metabotype-depleted	0	peripheral
K03795	high-metabotype-depleted	0	peripheral
K08963	high-metabotype-depleted	0	peripheral
K01708	high-metabotype-depleted	0	peripheral
K00483	high-metabotype-depleted	0	peripheral
K02815	high-metabotype-depleted	0	peripheral
K02771	high-metabotype-depleted	0	peripheral
K03274	high-metabotype-depleted	0	peripheral
K01876	high-metabotype-depleted	0	peripheral
K00423	high-metabotype-depleted	0	peripheral
K01867	high-metabotype-depleted	0	peripheral
K01051	high-metabotype-depleted	0	peripheral
K01132	high-metabotype-depleted	0	peripheral
K01209	high-metabotype-depleted	0	peripheral

K01887	high-metabotype-depleted	0	peripheral
K01784	high-metabotype-depleted	0	peripheral
K01627	high-metabotype-depleted	0	peripheral
K01880	high-metabotype-depleted	0	peripheral
K01515	high-metabotype-depleted	0	peripheral
K05934	high-metabotype-depleted	0	peripheral
K01042	high-metabotype-depleted	0	peripheral
K01883	high-metabotype-depleted	0	peripheral
K10985	high-metabotype-depleted	0	peripheral
K00257	high-metabotype-depleted	0	peripheral
K11191	high-metabotype-depleted	0	peripheral
K11192	high-metabotype-depleted	0	peripheral
K01890	high-metabotype-depleted	0	peripheral
K01451	high-metabotype-depleted	0	peripheral
K01001	high-metabotype-depleted	0	peripheral
K05780	high-metabotype-depleted	0	peripheral
K01866	high-metabotype-depleted	0	peripheral
K01869	high-metabotype-depleted	0	peripheral
K12554	high-metabotype-depleted	0	peripheral
K01119	high-metabotype-depleted	0	peripheral
K02259	high-metabotype-depleted	0	peripheral
K02190	high-metabotype-depleted	0	peripheral

K02377	high-metabotype-depleted	0	peripheral
K05396	high-metabotype-depleted	0	peripheral
K01881	high-metabotype-depleted	0	peripheral
K00881	high-metabotype-depleted	0	peripheral
K08092	high-metabotype-depleted	0	peripheral
K01873	high-metabotype-depleted	0	peripheral
K01761	high-metabotype-depleted	0	peripheral
K01868	high-metabotype-depleted	0	peripheral
K01813	high-metabotype-depleted	0	peripheral
K01779	high-metabotype-depleted	0	peripheral
K01442	high-metabotype-depleted	0	peripheral
K00096	high-metabotype-depleted	0	peripheral
K01870	high-metabotype-depleted	0	peripheral
K03390	high-metabotype-depleted	0	peripheral
K07806	high-metabotype-depleted	0	peripheral
K01889	high-metabotype-depleted	0	peripheral
K01950	high-metabotype-depleted	0	peripheral
K03269	high-metabotype-depleted	0	peripheral
K03404	high-metabotype-depleted	0	peripheral
K02745	high-metabotype-depleted	0	peripheral
K09516	high-metabotype-depleted	0	peripheral
K01906	high-metabotype-depleted	0	peripheral

K02169	high-metabotype-depleted	0	peripheral
K05275	high-metabotype-depleted	0	peripheral
K04720	high-metabotype-depleted	0	peripheral
K00019	high-metabotype-depleted	0	peripheral
K04568	high-metabotype-depleted	0	peripheral
K01844	high-metabotype-depleted	0	peripheral
K12308	high-metabotype-depleted	0	peripheral
K06045	high-metabotype-depleted	0	peripheral
K00805	high-metabotype-depleted	0	peripheral
K01684	high-metabotype-depleted	0	peripheral
K00086	high-metabotype-depleted	0	peripheral
K00972	high-metabotype-depleted	0	peripheral
K01879	high-metabotype-depleted	0	peripheral
K04567	high-metabotype-depleted	0	peripheral
K09019	high-metabotype-depleted	0	peripheral
K01201	high-metabotype-depleted	0	peripheral
K01564	high-metabotype-depleted	0	peripheral
K00043	high-metabotype-depleted	0	peripheral
K02809	high-metabotype-depleted	0	peripheral
K02810	high-metabotype-depleted	0	peripheral
K05297	high-metabotype-depleted	0	peripheral
K14338	high-metabotype-depleted	0	peripheral

K01872	high-metabotype-depleted	0	peripheral
K03366	high-metabotype-depleted	0	peripheral
K02567	high-metabotype-depleted	0	peripheral
K00090	high-metabotype-depleted	0	peripheral
K09759	high-metabotype-depleted	0	peripheral
K13921	high-metabotype-depleted	0	peripheral
K02763	high-metabotype-depleted	0	peripheral
K01112	high-metabotype-depleted	0	peripheral
K05305	high-metabotype-depleted	0	peripheral
K04566	high-metabotype-depleted	0	peripheral
K02610	high-metabotype-depleted	0	peripheral
K00082	high-metabotype-depleted	0	peripheral
K08678	high-metabotype-depleted	0	peripheral
K01101	high-metabotype-depleted	0	peripheral
K12527	high-metabotype-depleted	0	peripheral
K00980	high-metabotype-depleted	0	peripheral
K02764	high-metabotype-depleted	0	peripheral
K02765	high-metabotype-depleted	0	peripheral
K06151	high-metabotype-depleted	0	peripheral
K06152	high-metabotype-depleted	0	peripheral
K01590	high-metabotype-depleted	0	peripheral
K03392	high-metabotype-depleted	0	peripheral

K08679	high-metabotype-depleted	0	peripheral
K06282	high-metabotype-depleted	0	peripheral
K03388	high-metabotype-depleted	0	peripheral
K00020	high-metabotype-depleted	0	peripheral
K08261	high-metabotype-depleted	0	peripheral
K04022	high-metabotype-depleted	0	peripheral
K13043	high-metabotype-depleted	0	peripheral
K03862	high-metabotype-depleted	0	peripheral
K04091	high-metabotype-depleted	0	peripheral
K03635	high-metabotype-depleted	0	peripheral
K14164	Other	0	peripheral
K06166	Other	0	peripheral
K14188	Other	0	peripheral
K06879	Other	0	peripheral
K01199	Other	0	peripheral
K00836	Other	0	peripheral
K00462	Other	0	peripheral
K11196	Other	0	peripheral
K01916	Other	0	peripheral
K05712	Other	0	peripheral
K03332	Other	0	peripheral
K08358	Other	0	peripheral
K13019	Other	0	peripheral
K09461	Other	0	peripheral
K12528	Other	0	peripheral
K03389	Other	0	peripheral
K13566	Other	0	peripheral
K14465	Other	0	peripheral
K02746	Other	0	peripheral
K12452	Other	0	peripheral
K05601	Other	0	peripheral
K10986	Other	0	peripheral

K08969	Other	0	peripheral
K01210	Other	0	peripheral
K02747	Other	0	peripheral
K02813	Other	0	peripheral
K11646	Other	0	peripheral
K05395	Other	0	peripheral
K09882	Other	0	peripheral
K01574	Other	0	peripheral
K07310	Other	0	peripheral
K03405	Other	0	peripheral
K00119	Other	0	peripheral
K12529	Other	0	peripheral
K11214	Other	0	peripheral
K10984	Other	0	peripheral
K00373	Other	0	peripheral
K02808	Other	0	peripheral
K08357	Other	0	peripheral
K00370	Other	0	peripheral
K00371	Other	0	peripheral
K01718	Other	0	peripheral
K01044	Other	0	peripheral
K00130	Other	0	peripheral
K00934	Other	0	peripheral
K03863	Other	0	peripheral
K07309	high-metabotype-associated	0	peripheral
K02814	high-metabotype-associated	0	peripheral
K12661	high-metabotype-associated	0	peripheral
K08312	high-metabotype-associated	0	peripheral
K13774	high-metabotype-associated	0	peripheral
K02752	high-metabotype-associated	0	peripheral
K02753	high-metabotype-associated	0	peripheral
K06193	high-metabotype-associated	0	peripheral
K08351	high-metabotype-associated	0	peripheral

K04032	high-metabotype-associated	0	peripheral
K04480	high-metabotype-associated	0	peripheral
K00523	high-metabotype-associated	0	peripheral
K04103	high-metabotype-associated	0	peripheral
K00374	high-metabotype-associated	0	peripheral
K00372	high-metabotype-associated	0	peripheral
K06165	high-metabotype-associated	0	peripheral
K09835	high-metabotype-associated	0	peripheral
K05352	high-metabotype-associated	0	peripheral
K08096	high-metabotype-associated	0	peripheral
K00066	high-metabotype-associated	0	peripheral
K14080	high-metabotype-associated	0	peripheral
K14081	high-metabotype-associated	0	peripheral
K04835	high-metabotype-associated	0	peripheral
K00114	high-metabotype-associated	0	peripheral
K01725	high-metabotype-associated	0	peripheral
K01705	high-metabotype-associated	0	peripheral
K03636	high-metabotype-associated	0	peripheral
K03895	high-metabotype-enriched	0	peripheral
K00484	high-metabotype-enriched	0	peripheral
K05973	high-metabotype-enriched	0	peripheral
K00004	Other	0	peripheral
K00315	Other	0	peripheral

K00360	Other	0	peripheral
K00485	Other	0	peripheral
K00493	Other	0	peripheral
K00587	Other	0	peripheral
K00591	Other	0	peripheral
K00635	Other	0	peripheral
K00816	Other	0	peripheral
K00875	Other	0	peripheral
K00887	Other	0	peripheral
K00922	Other	0	peripheral
K01049	Other	0	peripheral
K01237	Other	0	peripheral
K01253	Other	0	peripheral
K01522	Other	0	peripheral
K02568	Other	0	peripheral
K02609	Other	0	peripheral
K02611	Other	0	peripheral
K02612	Other	0	peripheral
K02613	Other	0	peripheral
K03391	Other	0	peripheral
K03857	Other	0	peripheral
K04097	Other	0	peripheral
K04099	Other	0	peripheral
K06118	Other	0	peripheral
K06611	Other	0	peripheral
K07249	Other	0	peripheral
K07534	Other	0	peripheral
K07548	Other	0	peripheral
K10105	Other	0	peripheral
K11211	Other	0	peripheral
K11422	Other	0	peripheral
K11778	Other	0	peripheral
K12420	Other	0	peripheral
K12504	Other	0	peripheral
K13028	Other	0	peripheral
K13995	Other	0	peripheral
K14268	Other	0	peripheral
K14447	Other	0	peripheral
K14449	Other	0	peripheral
K08683	Other	4.00E-08	peripheral

K11147	high-metabotype-depleted	5.00E-08	peripheral
K11150	high-metabotype-depleted	5.00E-08	peripheral
K11153	high-metabotype-depleted	5.00E-08	peripheral
K11152	Other	5.00E-08	peripheral
K01746	high-metabotype-depleted	9.00E-08	peripheral
K03330	Other	9.00E-08	peripheral
K09482	high-metabotype-associated	9.00E-08	peripheral
K00147	high-metabotype-depleted	1.40E-07	peripheral
K02799	high-metabotype-depleted	1.70E-07	peripheral
K02800	high-metabotype-depleted	1.70E-07	peripheral
K02798	high-metabotype-depleted	1.70E-07	peripheral
K01488	high-metabotype-depleted	1.80E-07	peripheral
K01702	Other	1.80E-07	peripheral
K14267	high-metabotype-depleted	2.30E-07	peripheral
K13479	high-metabotype-depleted	2.80E-07	peripheral
K13481	high-metabotype-depleted	2.80E-07	peripheral
K03786	high-metabotype-depleted	2.80E-07	peripheral
K00087	high-metabotype-depleted	2.80E-07	peripheral
K03785	high-metabotype-depleted	2.80E-07	peripheral
K13480	Other	2.80E-07	peripheral
K13483	Other	2.80E-07	peripheral
K11178	high-metabotype-associated	2.80E-07	peripheral
K11177	high-metabotype-associated	2.80E-07	peripheral
K13482	high-metabotype-enriched	2.80E-07	peripheral

K00919	high-metabotype-depleted	3.50E-07	peripheral
K00014	high-metabotype-depleted	3.50E-07	peripheral
K01716	high-metabotype-depleted	3.50E-07	peripheral
K00841	high-metabotype-depleted	3.50E-07	peripheral
K08281	high-metabotype-depleted	4.00E-07	peripheral
K00985	high-metabotype-depleted	4.30E-07	peripheral
K01720	Other	4.40E-07	peripheral
K00932	Other	4.60E-07	peripheral
K00442	Other	4.60E-07	peripheral
K00441	Other	4.60E-07	peripheral
K00443	Other	4.60E-07	peripheral
K00440	high-metabotype-enriched	4.60E-07	peripheral
K06127	high-metabotype-depleted	4.70E-07	peripheral
K00891	high-metabotype-depleted	4.70E-07	peripheral
K00878	high-metabotype-depleted	6.10E-07	peripheral
K00703	high-metabotype-depleted	7.00E-07	peripheral
K01952	high-metabotype-depleted	7.00E-07	peripheral
K01937	high-metabotype-depleted	7.70E-07	peripheral
K01934	high-metabotype-depleted	8.40E-07	peripheral
K04561	high-metabotype-associated	1.03E-06	peripheral
K03421	high-metabotype-associated	1.05E-06	peripheral
K00400	high-metabotype-associated	1.05E-06	peripheral
K00399	high-metabotype-associated	1.05E-06	peripheral
K03422	high-metabotype-associated	1.05E-06	peripheral

K00401	high-metabotype-enriched	1.05E-06	peripheral
K00402	high-metabotype-enriched	1.05E-06	peripheral
K05887	high-metabotype-depleted	1.10E-06	peripheral
K01179	high-metabotype-depleted	1.33E-06	peripheral
K01225	high-metabotype-depleted	1.33E-06	peripheral
K00060	Other	1.41E-06	peripheral
K01951	high-metabotype-depleted	1.63E-06	peripheral
K05708	high-metabotype-associated	1.71E-06	peripheral
K05709	Other	1.71E-06	peripheral
K00133	high-metabotype-depleted	1.83E-06	peripheral
K03781	high-metabotype-depleted	1.86E-06	peripheral
K01496	high-metabotype-depleted	1.87E-06	peripheral
K05979	high-metabotype-depleted	1.97E-06	peripheral
K01941	Other	2.00E-06	peripheral
K07405	high-metabotype-depleted	2.06E-06	peripheral
K01176	high-metabotype-depleted	2.06E-06	peripheral
K01069	high-metabotype-depleted	2.37E-06	peripheral
K01588	high-metabotype-depleted	2.47E-06	peripheral
K01499	Other	2.50E-06	peripheral
K03779	high-metabotype-depleted	2.66E-06	peripheral
K03780	Other	2.66E-06	peripheral
K00059	high-metabotype-depleted	3.52E-06	peripheral
K02189	high-metabotype-depleted	3.52E-06	peripheral
K02191	high-metabotype-depleted	3.52E-06	peripheral

K01933	high-metabotype-depleted	3.72E-06	peripheral
K06863	high-metabotype-associated	3.87E-06	peripheral
K00882	high-metabotype-depleted	3.88E-06	peripheral
K08692	Other	3.89E-06	peripheral
K14067	Other	3.89E-06	peripheral
K00145	high-metabotype-depleted	4.48E-06	peripheral
K00053	high-metabotype-depleted	4.61E-06	peripheral
K00700	high-metabotype-depleted	5.79E-06	peripheral
K01465	high-metabotype-depleted	5.88E-06	peripheral
K05607	Other	6.37E-06	peripheral
K13766	Other	6.37E-06	peripheral
K12235	Other	7.24E-06	peripheral
K01816	high-metabotype-depleted	7.60E-06	peripheral
K00954	high-metabotype-depleted	7.69E-06	peripheral
K02201	high-metabotype-associated	7.69E-06	peripheral
K01576	high-metabotype-associated	7.89E-06	peripheral
K00645	high-metabotype-depleted	8.85E-06	peripheral
K13935	high-metabotype-enriched	8.85E-06	peripheral
K00290	high-metabotype-depleted	8.99E-06	peripheral
K09789	high-metabotype-depleted	9.79E-06	peripheral
K02170	high-metabotype-associated	9.79E-06	peripheral
K10536	high-metabotype-depleted	9.89E-06	peripheral
K04486	high-metabotype-depleted	9.96E-06	peripheral
K06446	high-metabotype-associated	1.03E-05	peripheral

K01703	high-metabotype-depleted	1.03E-05	peripheral
K01704	high-metabotype-depleted	1.03E-05	peripheral
K01826	Other	1.06E-05	peripheral
K00500	high-metabotype-depleted	1.10E-05	peripheral
K00941	high-metabotype-depleted	1.14E-05	peripheral
K00877	Other	1.14E-05	peripheral
K01945	high-metabotype-depleted	1.31E-05	peripheral
K01935	high-metabotype-depleted	1.36E-05	peripheral
K01487	high-metabotype-depleted	1.45E-05	peripheral
K13923	high-metabotype-depleted	1.50E-05	peripheral
K00852	high-metabotype-depleted	1.55E-05	peripheral
K07251	high-metabotype-depleted	1.56E-05	peripheral
K02340	high-metabotype-depleted	1.57E-05	peripheral
K02342	high-metabotype-depleted	1.57E-05	peripheral
K02341	high-metabotype-depleted	1.57E-05	peripheral
K02337	high-metabotype-depleted	1.57E-05	peripheral
K02335	high-metabotype-depleted	1.57E-05	peripheral
K02338	high-metabotype-depleted	1.57E-05	peripheral
K02343	high-metabotype-depleted	1.57E-05	peripheral
K03763	high-metabotype-depleted	1.57E-05	peripheral
K02345	Other	1.57E-05	peripheral
K02344	Other	1.57E-05	peripheral
K02319	high-metabotype-associated	1.57E-05	peripheral

K02339	high-metabotype-associated	1.57E-05	peripheral
K02323	high-metabotype-associated	1.57E-05	peripheral
K02322	high-metabotype-associated	1.57E-05	peripheral
K14159	high-metabotype-enriched	1.57E-05	peripheral
K02324	Other	1.57E-05	peripheral
K02327	Other	1.57E-05	peripheral
K01071	high-metabotype-depleted	1.62E-05	peripheral
K00835	Other	1.65E-05	peripheral
K00872	high-metabotype-depleted	1.68E-05	peripheral
K02204	high-metabotype-depleted	1.68E-05	peripheral
K01486	high-metabotype-depleted	1.70E-05	peripheral
K00287	high-metabotype-depleted	1.75E-05	peripheral
K13938	Other	1.75E-05	peripheral
K00689	high-metabotype-depleted	1.80E-05	peripheral
K05341	high-metabotype-depleted	1.80E-05	peripheral
K03185	Other	1.85E-05	peripheral
K10805	high-metabotype-depleted	1.90E-05	peripheral
K10806	high-metabotype-depleted	1.90E-05	peripheral
K10804	high-metabotype-depleted	1.90E-05	peripheral
K01874	high-metabotype-depleted	2.20E-05	peripheral
K03562	high-metabotype-depleted	2.34E-05	peripheral
K01220	Other	2.35E-05	peripheral
K07516	high-metabotype-depleted	2.40E-05	peripheral
K07256	Other	2.42E-05	peripheral
K06120	high-metabotype-depleted	2.47E-05	peripheral

K06122	high-metabotype-depleted	2.47E-05	peripheral
K06121	Other	2.47E-05	peripheral
K01509	Other	2.50E-05	peripheral
K00074	high-metabotype-depleted	2.56E-05	peripheral
K12255	Other	2.57E-05	peripheral
K00216	high-metabotype-depleted	2.61E-05	peripheral
K00297	high-metabotype-depleted	2.65E-05	peripheral
K01712	high-metabotype-depleted	2.67E-05	peripheral
K00904	Other	2.83E-05	peripheral
K01707	Other	2.91E-05	peripheral
K02371	high-metabotype-depleted	2.97E-05	peripheral
K10780	high-metabotype-associated	2.97E-05	peripheral
K01893	high-metabotype-depleted	3.06E-05	peripheral
K01560	high-metabotype-depleted	3.09E-05	peripheral
K01561	high-metabotype-depleted	3.09E-05	peripheral
K00800	high-metabotype-depleted	3.25E-05	peripheral
K01057	high-metabotype-depleted	3.26E-05	peripheral
K07404	high-metabotype-depleted	3.26E-05	peripheral
K02819	high-metabotype-depleted	3.30E-05	peripheral
K02818	high-metabotype-depleted	3.30E-05	peripheral
K02817	Other	3.30E-05	peripheral
K00895	high-metabotype-depleted	3.39E-05	peripheral
K00819	high-metabotype-depleted	3.41E-05	peripheral
K00647	high-metabotype-depleted	3.54E-05	peripheral

K09458	high-metabotype-depleted	3.54E-05	peripheral
K02303	high-metabotype-depleted	3.59E-05	peripheral
K02496	Other	3.59E-05	peripheral
K01792	high-metabotype-depleted	3.71E-05	peripheral
K01770	high-metabotype-depleted	3.85E-05	peripheral
K11176	Other	3.85E-05	peripheral
K01502	high-metabotype-depleted	3.85E-05	peripheral
K00224	Other	3.92E-05	peripheral
K01908	high-metabotype-associated	4.15E-05	peripheral
K03336	high-metabotype-depleted	4.17E-05	peripheral
K02803	Other	4.17E-05	peripheral
K02804	Other	4.17E-05	peripheral
K02802	high-metabotype-associated	4.17E-05	peripheral
K14379	high-metabotype-depleted	4.18E-05	peripheral
K03841	high-metabotype-depleted	4.20E-05	peripheral
K04041	high-metabotype-depleted	4.20E-05	peripheral
K02446	high-metabotype-depleted	4.20E-05	peripheral
K00137	high-metabotype-associated	4.21E-05	peripheral
K00833	high-metabotype-depleted	4.24E-05	peripheral
K00927	high-metabotype-depleted	4.25E-05	peripheral
K00885	Other	4.57E-05	peripheral
K01838	high-metabotype-depleted	4.62E-05	peripheral
K00863	high-metabotype-depleted	4.63E-05	peripheral
K05878	high-metabotype-depleted	4.63E-05	peripheral

K05879	high-metabotype-depleted	4.63E-05	peripheral
K01611	high-metabotype-depleted	4.85E-05	peripheral
K01856	Other	5.26E-05	peripheral
K05342	Other	5.32E-05	peripheral
K01424	high-metabotype-depleted	5.34E-05	peripheral
K01953	high-metabotype-depleted	5.34E-05	peripheral
K01914	high-metabotype-depleted	5.34E-05	peripheral
K12658	high-metabotype-depleted	5.46E-05	peripheral
K01907	high-metabotype-enriched	5.50E-05	peripheral
K01968	Other	5.72E-05	peripheral
K01969	high-metabotype-associated	5.72E-05	peripheral
K00134	high-metabotype-depleted	5.77E-05	peripheral
K00150	Other	5.77E-05	peripheral
K05298	high-metabotype-enriched	5.77E-05	peripheral
K10705	Other	5.77E-05	peripheral
K00558	high-metabotype-depleted	5.78E-05	peripheral
K01095	high-metabotype-depleted	5.80E-05	peripheral
K01096	Other	5.80E-05	peripheral
K08352	high-metabotype-depleted	5.83E-05	peripheral
K08354	high-metabotype-depleted	5.83E-05	peripheral
K08353	Other	5.83E-05	peripheral
K01241	high-metabotype-depleted	5.98E-05	peripheral
K03417	high-metabotype-depleted	6.06E-05	peripheral
K01008	high-metabotype-depleted	6.20E-05	peripheral
K09471	Other	6.29E-05	peripheral

K02775	high-metabotype-depleted	6.51E-05	peripheral
K02774	high-metabotype-depleted	6.51E-05	peripheral
K02773	high-metabotype-depleted	6.51E-05	peripheral
K00253	high-metabotype-depleted	6.52E-05	peripheral
K01817	high-metabotype-depleted	6.53E-05	peripheral
K00042	high-metabotype-depleted	6.53E-05	peripheral
K00691	high-metabotype-depleted	6.64E-05	peripheral
K02439	high-metabotype-depleted	6.72E-05	peripheral
K07246	high-metabotype-associated	6.82E-05	peripheral
K03475	high-metabotype-depleted	6.95E-05	peripheral
K02821	high-metabotype-depleted	6.95E-05	peripheral
K02822	Other	6.95E-05	peripheral
K02594	high-metabotype-depleted	7.12E-05	peripheral
K05351	high-metabotype-depleted	7.15E-05	peripheral
K01857	Other	7.18E-05	peripheral
K02793	high-metabotype-depleted	7.21E-05	peripheral
K02795	high-metabotype-depleted	7.21E-05	peripheral
K02794	high-metabotype-depleted	7.21E-05	peripheral
K02796	high-metabotype-depleted	7.21E-05	peripheral
K00094	high-metabotype-depleted	7.27E-05	peripheral
K00693	high-metabotype-depleted	7.48E-05	peripheral
K01608	Other	7.53E-05	peripheral
K05825	Other	7.58E-05	peripheral

K01800	high-metabotype-depleted	7.70E-05	peripheral
K01520	high-metabotype-depleted	7.82E-05	peripheral
K00957	high-metabotype-depleted	8.08E-05	peripheral
K00956	high-metabotype-depleted	8.08E-05	peripheral
K00958	high-metabotype-enriched	8.08E-05	peripheral
K04098	Other	8.09E-05	peripheral
K09722	high-metabotype-enriched	8.23E-05	peripheral
K01715	high-metabotype-depleted	8.26E-05	peripheral
K01598	high-metabotype-depleted	8.40E-05	peripheral
K01615	high-metabotype-depleted	8.52E-05	peripheral
K00641	high-metabotype-depleted	8.57E-05	peripheral
K03707	high-metabotype-depleted	8.90E-05	peripheral
K00467	Other	9.01E-05	peripheral
K11263	high-metabotype-depleted	9.06E-05	peripheral
K01523	high-metabotype-depleted	9.17E-05	peripheral
K06034	high-metabotype-associated	9.19E-05	peripheral
K13039	high-metabotype-enriched	9.19E-05	peripheral
K00460	Other	9.26E-05	peripheral
K11264	high-metabotype-depleted	9.27E-05	peripheral
K10817	Other	9.27E-05	peripheral
K01609	high-metabotype-depleted	9.47E-05	peripheral
K00451	high-metabotype-enriched	9.49E-05	peripheral
K00639	high-metabotype-depleted	9.53E-05	peripheral

K04516	high-metabotype-depleted	9.57E-05	peripheral
K01850	high-metabotype-depleted	9.57E-05	peripheral
K06209	high-metabotype-depleted	9.57E-05	peripheral
K04092	Other	9.57E-05	peripheral
K04093	Other	9.57E-05	peripheral
K06208	high-metabotype-associated	9.57E-05	peripheral
K00088	high-metabotype-depleted	9.65E-05	peripheral
K06982	Other	9.70E-05	peripheral
K01821	Other	9.73E-05	peripheral
K00651	high-metabotype-depleted	9.81E-05	peripheral
K00938	Other	0.00010267	peripheral
K01785	high-metabotype-depleted	0.00010448	peripheral
K03473	high-metabotype-depleted	0.00011018	peripheral
K10206	high-metabotype-depleted	0.00011088	peripheral
K01591	high-metabotype-depleted	0.0001112	peripheral
K00241	high-metabotype-depleted	0.00011409	peripheral
K00240	high-metabotype-depleted	0.00011409	peripheral
K00246	high-metabotype-depleted	0.00011409	peripheral
K00235	high-metabotype-depleted	0.00011409	peripheral
K00245	high-metabotype-depleted	0.00011409	peripheral
K00239	high-metabotype-depleted	0.00011409	peripheral
K00244	high-metabotype-depleted	0.00011409	peripheral
K00247	Other	0.00011409	peripheral
K00242	Other	0.00011409	peripheral
K00234	Other	0.00011409	peripheral

K01834	high-metabotype-depleted	0.00011417	peripheral
K00943	high-metabotype-depleted	0.00011544	peripheral
K03426	high-metabotype-depleted	0.00011656	peripheral
K01464	high-metabotype-depleted	0.00011881	peripheral
K01457	high-metabotype-depleted	0.00012255	peripheral
K01473	high-metabotype-depleted	0.00012279	peripheral
K01474	high-metabotype-depleted	0.00012279	peripheral
K01847	high-metabotype-depleted	0.00012531	peripheral
K01848	high-metabotype-depleted	0.00012531	peripheral
K01849	Other	0.00012531	peripheral
K01687	high-metabotype-depleted	0.00012546	peripheral
K00453	high-metabotype-depleted	0.00012871	peripheral
K00003	high-metabotype-depleted	0.00013532	peripheral
K01028	high-metabotype-depleted	0.00013536	peripheral
K01029	Other	0.00013536	peripheral
K01027	Other	0.00013536	peripheral
K03021	high-metabotype-depleted	0.0001356	peripheral
K03040	high-metabotype-depleted	0.0001356	peripheral
K03048	high-metabotype-depleted	0.0001356	peripheral
K03046	high-metabotype-depleted	0.0001356	peripheral
K03044	high-metabotype-depleted	0.0001356	peripheral
K03043	high-metabotype-depleted	0.0001356	peripheral
K03010	high-metabotype-depleted	0.0001356	peripheral

K03050	high-metabotype-depleted	0.0001356	peripheral
K03053	high-metabotype-depleted	0.0001356	peripheral
K13797	high-metabotype-depleted	0.0001356	peripheral
K03060	high-metabotype-depleted	0.0001356	peripheral
K03042	Other	0.0001356	peripheral
K03006	Other	0.0001356	peripheral
K02999	Other	0.0001356	peripheral
K03057	Other	0.0001356	peripheral
K03051	Other	0.0001356	peripheral
K03055	Other	0.0001356	peripheral
K13798	Other	0.0001356	peripheral
K03018	high-metabotype-associated	0.0001356	peripheral
K03056	high-metabotype-associated	0.0001356	peripheral
K03045	high-metabotype-associated	0.0001356	peripheral
K03047	high-metabotype-associated	0.0001356	peripheral
K03041	high-metabotype-associated	0.0001356	peripheral
K03049	high-metabotype-enriched	0.0001356	peripheral
K03002	Other	0.0001356	peripheral
K03027	Other	0.0001356	peripheral
K03058	Other	0.0001356	peripheral
K03852	high-metabotype-depleted	0.00013623	peripheral
K01804	high-metabotype-depleted	0.00013664	peripheral
K00532	high-metabotype-depleted	0.00014169	peripheral
K13950	high-metabotype-depleted	0.00014169	peripheral
K01665	high-metabotype-depleted	0.00014169	peripheral
K00533	high-metabotype-depleted	0.00014169	peripheral

K01664	high-metabotype-depleted	0.00014169	peripheral
K06441	Other	0.00014169	peripheral
K00534	Other	0.00014169	peripheral
K10796	high-metabotype-depleted	0.00014213	peripheral
K10795	high-metabotype-depleted	0.00014213	peripheral
K10793	high-metabotype-depleted	0.00014213	peripheral
K10794	Other	0.00014213	peripheral
K01753	high-metabotype-depleted	0.00014281	peripheral
K02787	Other	0.00014294	peripheral
K02788	Other	0.00014294	peripheral
K02786	Other	0.00014294	peripheral
K00101	Other	0.00014388	peripheral
K05993	high-metabotype-depleted	0.00014401	peripheral
K01252	high-metabotype-depleted	0.00014401	peripheral
K00186	high-metabotype-depleted	0.0001449	peripheral
K00187	Other	0.0001449	peripheral
K00188	high-metabotype-associated	0.0001449	peripheral
K01771	Other	0.00014647	peripheral
K02438	high-metabotype-depleted	0.00014689	peripheral
K01236	Other	0.00014689	peripheral
K01491	high-metabotype-depleted	0.00014693	peripheral
K13403	Other	0.00014693	peripheral
K03476	high-metabotype-depleted	0.00014975	peripheral
K00385	high-metabotype-depleted	0.00015059	peripheral
K00381	Other	0.00015059	peripheral
K00380	Other	0.00015059	peripheral
K00364	high-metabotype-depleted	0.00015716	peripheral
K00050	high-metabotype-depleted	0.00015856	peripheral

K00975	high-metabotype-depleted	0.00015998	peripheral
K01955	high-metabotype-depleted	0.00016086	peripheral
K01956	high-metabotype-depleted	0.00016086	peripheral
K01954	high-metabotype-depleted	0.00016086	peripheral
K00077	high-metabotype-depleted	0.00016109	peripheral
K00005	high-metabotype-depleted	0.00016174	peripheral
K12251	high-metabotype-depleted	0.00016288	peripheral
K01478	Other	0.00016447	peripheral
K14446	Other	0.00016731	peripheral
K01493	high-metabotype-depleted	0.00016733	peripheral
K00207	high-metabotype-depleted	0.0001703	peripheral
K00146	high-metabotype-associated	0.00017234	peripheral
K01494	high-metabotype-depleted	0.00017296	peripheral
K00023	high-metabotype-depleted	0.00017311	peripheral
K00705	high-metabotype-depleted	0.00017544	peripheral
K01699	high-metabotype-depleted	0.00017642	peripheral
K13919	Other	0.00017642	peripheral
K13920	Other	0.00017642	peripheral
K01775	high-metabotype-depleted	0.00017944	peripheral
K00599	high-metabotype-depleted	0.0001809	peripheral
K07732	Other	0.00018115	peripheral
K00861	Other	0.00018115	peripheral
K00860	high-metabotype-depleted	0.00018259	peripheral
K03153	Other	0.00018341	peripheral
K00798	high-metabotype-depleted	0.00018927	peripheral

K00102	high-metabotype-depleted	0.00018927	peripheral
K02435	high-metabotype-depleted	0.00018951	peripheral
K02434	high-metabotype-depleted	0.00018951	peripheral
K02433	high-metabotype-depleted	0.00018951	peripheral
K10783	high-metabotype-depleted	0.00019185	peripheral
K01087	Other	0.00019262	peripheral
K00013	high-metabotype-depleted	0.00019271	peripheral
K05915	Other	0.00019308	peripheral
K01845	high-metabotype-depleted	0.00019379	peripheral
K13942	Other	0.00019615	peripheral
K00634	high-metabotype-depleted	0.00019729	peripheral
K01896	Other	0.00019729	peripheral
K09903	high-metabotype-depleted	0.00019889	peripheral
K05343	high-metabotype-depleted	0.00020036	intermediate
K01692	high-metabotype-depleted	0.00020071	intermediate
K00499	high-metabotype-associated	0.00020289	intermediate
K00255	high-metabotype-depleted	0.00020483	intermediate
K07512	Other	0.00020483	intermediate
K04487	high-metabotype-depleted	0.00020498	intermediate
K00024	high-metabotype-depleted	0.00020515	intermediate
K00051	high-metabotype-depleted	0.00020515	intermediate
K00116	high-metabotype-enriched	0.00020515	intermediate
K00025	Other	0.00020515	intermediate
K00026	Other	0.00020515	intermediate
K11381	high-metabotype-depleted	0.00020527	intermediate

K00166	Other	0.00020527	intermediate
K00167	Other	0.00020527	intermediate
K00472	high-metabotype-depleted	0.00020572	intermediate
K00655	high-metabotype-depleted	0.00020602	intermediate
K13509	high-metabotype-depleted	0.00020602	intermediate
K00856	high-metabotype-depleted	0.00021039	intermediate
K01438	high-metabotype-depleted	0.00021304	intermediate
K00899	high-metabotype-depleted	0.00021523	intermediate
K00143	high-metabotype-depleted	0.00021869	intermediate
K01607	high-metabotype-depleted	0.00021954	intermediate
K03081	Other	0.00022225	intermediate
K01578	high-metabotype-depleted	0.00022287	intermediate
K09459	high-metabotype-depleted	0.00022568	intermediate
K01858	high-metabotype-depleted	0.00022644	intermediate
K01750	high-metabotype-depleted	0.00022844	intermediate
K00995	high-metabotype-depleted	0.00022884	intermediate
K00640	high-metabotype-depleted	0.00023171	intermediate
K03815	high-metabotype-depleted	0.00023428	intermediate
K02492	high-metabotype-depleted	0.00023529	intermediate
K00252	Other	0.00023694	intermediate
K01613	high-metabotype-depleted	0.00023794	intermediate
K09018	Other	0.00023998	intermediate
K09024	Other	0.00023998	intermediate
K00151	high-metabotype-depleted	0.00024344	intermediate
K10219	Other	0.00024344	intermediate

K00945	high-metabotype-depleted	0.00024417	intermediate
K13800	Other	0.00024417	intermediate
K00762	high-metabotype-depleted	0.00024478	intermediate
K11755	high-metabotype-depleted	0.00024876	intermediate
K03735	high-metabotype-depleted	0.00025504	intermediate
K03736	high-metabotype-depleted	0.00025504	intermediate
K04019	Other	0.00025504	intermediate
K10527	high-metabotype-depleted	0.00025543	intermediate
K03119	high-metabotype-associated	0.00025585	intermediate
K00931	high-metabotype-depleted	0.00026041	intermediate
K01966	high-metabotype-depleted	0.00026172	intermediate
K01965	Other	0.00026172	intermediate
K02626	high-metabotype-depleted	0.00026237	intermediate
K01585	high-metabotype-depleted	0.00026237	intermediate
K01584	high-metabotype-depleted	0.00026237	intermediate
K01583	Other	0.00026237	intermediate
K00610	high-metabotype-depleted	0.00026719	intermediate
K00609	high-metabotype-depleted	0.00026719	intermediate
K02500	high-metabotype-depleted	0.00027576	intermediate
K02501	high-metabotype-depleted	0.00027576	intermediate
K01663	high-metabotype-associated	0.00027576	intermediate
K00226	high-metabotype-depleted	0.00028187	intermediate
K05713	Other	0.00028252	intermediate
K07031	high-metabotype-depleted	0.00028355	intermediate

K00030	high-metabotype-depleted	0.00028381	intermediate
K03430	Other	0.0002839	intermediate
K09469	high-metabotype-associated	0.0002839	intermediate
K01745	high-metabotype-depleted	0.00028449	intermediate
K01183	high-metabotype-depleted	0.00028461	intermediate
K13381	high-metabotype-associated	0.00028461	intermediate
K01689	high-metabotype-depleted	0.000285	intermediate
K00818	high-metabotype-depleted	0.00028502	intermediate
K00854	high-metabotype-depleted	0.00028611	intermediate
K00547	high-metabotype-depleted	0.00029142	intermediate
K01679	high-metabotype-depleted	0.00029149	intermediate
K01676	high-metabotype-depleted	0.00029149	intermediate
K01678	high-metabotype-depleted	0.00029149	intermediate
K01677	high-metabotype-depleted	0.00029149	intermediate
K03379	high-metabotype-depleted	0.00029342	intermediate
K09021	high-metabotype-depleted	0.00029533	intermediate
K03462	high-metabotype-depleted	0.00029648	intermediate
K01897	high-metabotype-depleted	0.00029986	intermediate
K00955	high-metabotype-depleted	0.00030498	intermediate
K13811	high-metabotype-enriched	0.00030498	intermediate
K01706	high-metabotype-depleted	0.0003118	intermediate
K01617	Other	0.00032071	intermediate
K00549	high-metabotype-depleted	0.00032118	intermediate

K01917	high-metabotype-depleted	0.00033709	intermediate
K01460	high-metabotype-depleted	0.00033709	intermediate
K01597	Other	0.00033922	intermediate
K01814	high-metabotype-depleted	0.00034177	intermediate
K03184	high-metabotype-associated	0.00034493	intermediate
K06134	Other	0.00034493	intermediate
K00763	high-metabotype-depleted	0.00035282	intermediate
K04020	Other	0.00035312	intermediate
K01823	high-metabotype-associated	0.00035481	intermediate
K00928	high-metabotype-depleted	0.00035483	intermediate
K01115	Other	0.00035662	intermediate
K13922	Other	0.00035751	intermediate
K00883	high-metabotype-depleted	0.00035865	intermediate
K02560	high-metabotype-depleted	0.00035966	intermediate
K01673	high-metabotype-depleted	0.00036085	intermediate
K01674	Other	0.00036085	intermediate
K00951	high-metabotype-depleted	0.00036186	intermediate
K07816	high-metabotype-depleted	0.00036186	intermediate
K00757	high-metabotype-depleted	0.00036817	intermediate
K00248	high-metabotype-depleted	0.00036872	intermediate
K09478	Other	0.00036872	intermediate
K03335	high-metabotype-depleted	0.0003737	intermediate
K13763	Other	0.00037462	intermediate
K14084	high-metabotype-depleted	0.0003783	intermediate
K14083	high-metabotype-associated	0.0003783	intermediate
K14082	Other	0.0003783	intermediate

K03526	high-metabotype-depleted	0.00037858	intermediate
K09020	Other	0.00037939	intermediate
K00756	high-metabotype-depleted	0.00037942	intermediate
K00016	high-metabotype-depleted	0.00037947	intermediate
K11261	Other	0.00038719	intermediate
K00202	Other	0.00038719	intermediate
K11260	Other	0.00038719	intermediate
K00200	high-metabotype-associated	0.00038719	intermediate
K00203	high-metabotype-associated	0.00038719	intermediate
K00205	high-metabotype-associated	0.00038719	intermediate
K00201	high-metabotype-associated	0.00038719	intermediate
K00204	high-metabotype-associated	0.00038719	intermediate
K01923	high-metabotype-depleted	0.0003876	intermediate
K01728	high-metabotype-depleted	0.00038909	intermediate
K01731	Other	0.00038909	intermediate
K09472	high-metabotype-associated	0.00039059	intermediate
K01776	high-metabotype-depleted	0.00039681	intermediate
K00286	high-metabotype-depleted	0.00040025	intermediate
K00210	high-metabotype-depleted	0.0004052	intermediate
K04517	high-metabotype-depleted	0.0004052	intermediate
K01589	high-metabotype-depleted	0.00041197	intermediate
K00536	Other	0.00041292	intermediate
K02790	high-metabotype-depleted	0.00041846	intermediate
K02791	high-metabotype-depleted	0.00041846	intermediate

K01632	high-metabotype-depleted	0.00041909	intermediate
K00052	high-metabotype-depleted	0.00042582	intermediate
K01620	high-metabotype-depleted	0.00042586	intermediate
K02778	high-metabotype-depleted	0.00043344	intermediate
K02779	high-metabotype-depleted	0.00043344	intermediate
K03078	high-metabotype-associated	0.0004366	intermediate
K05884	high-metabotype-depleted	0.00044206	intermediate
K03394	high-metabotype-depleted	0.0004463	intermediate
K02304	high-metabotype-depleted	0.0004463	intermediate
K01091	high-metabotype-depleted	0.0004467	intermediate
K06132	high-metabotype-depleted	0.0004471	intermediate
K06131	high-metabotype-depleted	0.0004471	intermediate
K13498	high-metabotype-depleted	0.00044764	intermediate
K00432	high-metabotype-depleted	0.00044944	intermediate
K00318	high-metabotype-depleted	0.00045066	intermediate
K11788	Other	0.00045283	intermediate
K01768	high-metabotype-depleted	0.00045429	intermediate
K05873	Other	0.00045429	intermediate
K05851	high-metabotype-associated	0.00045429	intermediate
K11029	Other	0.00045429	intermediate
K05362	high-metabotype-depleted	0.00046408	intermediate
K00925	high-metabotype-depleted	0.00046817	intermediate
K01902	high-metabotype-depleted	0.00047	intermediate

K01903	high-metabotype-depleted	0.00047	intermediate
K01899	Other	0.00047	intermediate
K01900	Other	0.00047	intermediate
K12657	high-metabotype-depleted	0.00047036	intermediate
K03778	high-metabotype-depleted	0.00047181	intermediate
K03777	high-metabotype-depleted	0.00047181	intermediate
K00657	high-metabotype-depleted	0.00048159	intermediate
K00108	high-metabotype-depleted	0.00048287	intermediate
K11440	high-metabotype-depleted	0.00048287	intermediate
K01783	high-metabotype-depleted	0.0004873	intermediate
K02554	Other	0.00049075	intermediate
K00568	high-metabotype-depleted	0.00049168	intermediate
K00449	high-metabotype-depleted	0.00049431	intermediate
K00448	Other	0.00049431	intermediate
K00606	high-metabotype-depleted	0.0004987	intermediate
K05364	high-metabotype-depleted	0.00050302	intermediate
K00293	Other	0.00050452	intermediate
K01875	high-metabotype-depleted	0.00050616	intermediate
K01480	high-metabotype-depleted	0.00050887	intermediate
K00078	high-metabotype-depleted	0.00050952	intermediate
K00075	high-metabotype-depleted	0.00050982	intermediate
K00917	Other	0.00051095	intermediate
K11517	high-metabotype-depleted	0.00051289	intermediate
K00104	high-metabotype-depleted	0.00051289	intermediate

K11473	high-metabotype-depleted	0.00051289	intermediate
K11472	Other	0.00051289	intermediate
K01479	high-metabotype-depleted	0.00051379	intermediate
K02536	high-metabotype-depleted	0.00051648	intermediate
K01734	high-metabotype-depleted	0.0005281	intermediate
K01788	high-metabotype-depleted	0.00052884	intermediate
K00991	high-metabotype-depleted	0.000529	intermediate
K01913	high-metabotype-depleted	0.00053081	intermediate
K10213	high-metabotype-associated	0.00053949	intermediate
K00259	high-metabotype-depleted	0.00054203	intermediate
K01592	high-metabotype-depleted	0.00054318	intermediate
K11808	Other	0.00054646	intermediate
K00930	high-metabotype-depleted	0.00054922	intermediate
K06989	high-metabotype-depleted	0.00055189	intermediate
K00008	high-metabotype-depleted	0.00055238	intermediate
K09758	high-metabotype-depleted	0.00055547	intermediate
K00368	Other	0.00055707	intermediate
K00849	high-metabotype-depleted	0.00056058	intermediate
K05823	high-metabotype-depleted	0.00056143	intermediate
K05936	high-metabotype-depleted	0.00056429	intermediate
K00611	high-metabotype-depleted	0.0005662	intermediate
K02232	high-metabotype-depleted	0.00056781	intermediate
K11752	high-metabotype-depleted	0.00056781	intermediate

K00900	high-metabotype-enriched	0.00056781	intermediate
K01498	Other	0.00056781	intermediate
K01659	high-metabotype-depleted	0.00056787	intermediate
K10675	Other	0.00056795	intermediate
K00889	high-metabotype-depleted	0.00056816	intermediate
K01524	high-metabotype-depleted	0.00056816	intermediate
K01514	high-metabotype-depleted	0.00056816	intermediate
K01843	high-metabotype-depleted	0.00056851	intermediate
K03273	high-metabotype-depleted	0.00056851	intermediate
K05363	high-metabotype-depleted	0.00056851	intermediate
K01103	high-metabotype-enriched	0.00056851	intermediate
K01455	Other	0.00056866	intermediate
K01423	high-metabotype-depleted	0.00056886	intermediate
K06013	Other	0.00056886	intermediate
K10220	Other	0.00056886	intermediate
K08693	Other	0.00056921	intermediate
K01759	high-metabotype-depleted	0.00056947	intermediate
K13018	high-metabotype-depleted	0.00056992	intermediate
K05603	Other	0.00057085	intermediate
K01458	high-metabotype-depleted	0.00057156	intermediate
K05822	high-metabotype-associated	0.00057418	intermediate
K03151	high-metabotype-depleted	0.00057564	intermediate
K01639	high-metabotype-depleted	0.00057647	intermediate
K01468	high-metabotype-depleted	0.00057659	intermediate
K11212	high-metabotype-associated	0.00057707	intermediate

K00793	high-metabotype-depleted	0.00057886	intermediate
K00376	high-metabotype-enriched	0.00057887	intermediate
K00604	high-metabotype-depleted	0.00058106	intermediate
K01048	high-metabotype-depleted	0.00058915	intermediate
K01126	high-metabotype-depleted	0.00058985	intermediate
K01079	high-metabotype-depleted	0.00059566	intermediate
K01654	high-metabotype-depleted	0.00060501	intermediate
K05304	high-metabotype-enriched	0.00060501	intermediate
K01187	high-metabotype-depleted	0.00061455	intermediate
K12316	Other	0.00061455	intermediate
K07130	high-metabotype-depleted	0.00061689	intermediate
K00799	Other	0.00062447	intermediate
K01226	high-metabotype-depleted	0.00062478	intermediate
K04518	high-metabotype-depleted	0.00062574	intermediate
K01713	Other	0.00062574	intermediate
K00217	high-metabotype-associated	0.00062701	intermediate
K01556	high-metabotype-associated	0.00063113	intermediate
K01924	high-metabotype-depleted	0.0006324	intermediate
K01682	high-metabotype-depleted	0.00063305	intermediate
K00135	Other	0.00064553	intermediate
K01958	high-metabotype-depleted	0.00065735	intermediate
K01960	high-metabotype-depleted	0.00065735	intermediate
K01959	high-metabotype-depleted	0.00065735	intermediate

K05921	high-metabotype-depleted	0.00065755	intermediate
K10764	high-metabotype-depleted	0.00066414	intermediate
K01733	high-metabotype-depleted	0.00066586	intermediate
K00058	high-metabotype-depleted	0.00066587	intermediate
K08093	high-metabotype-depleted	0.00067533	intermediate
K00430	Other	0.00067583	intermediate
K02783	high-metabotype-depleted	0.00067775	intermediate
K02781	high-metabotype-depleted	0.00067775	intermediate
K02782	high-metabotype-depleted	0.00067775	intermediate
K00952	high-metabotype-depleted	0.00068281	intermediate
K00969	high-metabotype-depleted	0.00068281	intermediate
K13522	Other	0.00068281	intermediate
K00220	Other	0.000683	intermediate
K00109	Other	0.00068834	intermediate
K00826	high-metabotype-depleted	0.00069085	intermediate
K00263	Other	0.00069085	intermediate
K01810	high-metabotype-depleted	0.00069089	intermediate
K06859	high-metabotype-depleted	0.00069089	intermediate
K08097	high-metabotype-associated	0.00069483	intermediate
K00892	high-metabotype-depleted	0.00070238	intermediate
K11781	Other	0.00070467	intermediate
K11780	high-metabotype-associated	0.00070467	intermediate
K01581	high-metabotype-depleted	0.0007072	intermediate
K01040	high-metabotype-depleted	0.00070868	intermediate

K01039	high-metabotype-depleted	0.00070868	intermediate
K01928	high-metabotype-depleted	0.00071116	intermediate
K01489	high-metabotype-depleted	0.00071919	intermediate
K01922	high-metabotype-depleted	0.00072095	intermediate
K01075	Other	0.00072111	intermediate
K01693	high-metabotype-depleted	0.00072365	intermediate
K01819	Other	0.00072674	intermediate
K07173	high-metabotype-depleted	0.00072803	intermediate
K01619	high-metabotype-depleted	0.00073033	intermediate
K01726	high-metabotype-depleted	0.0007463	intermediate
K01841	high-metabotype-depleted	0.00075556	intermediate
K08260	Other	0.00075602	intermediate
K03782	high-metabotype-associated	0.00076074	intermediate
K06133	high-metabotype-depleted	0.00076247	intermediate
K00997	high-metabotype-depleted	0.00076247	intermediate
K00034	high-metabotype-depleted	0.00076828	intermediate
K01433	high-metabotype-depleted	0.00077054	intermediate
K00858	high-metabotype-depleted	0.0007732	intermediate
K00324	high-metabotype-depleted	0.0007732	intermediate
K00325	Other	0.0007732	intermediate
K00322	Other	0.0007732	intermediate
K00323	Other	0.0007732	intermediate
K11175	high-metabotype-depleted	0.00078603	intermediate
K08289	high-metabotype-depleted	0.00078603	intermediate
K00601	Other	0.00078603	intermediate

K05551	high-metabotype-depleted	0.00079068	intermediate
K05552	Other	0.00079068	intermediate
K06215	high-metabotype-depleted	0.00079498	intermediate
K08681	high-metabotype-depleted	0.00079498	intermediate
K03337	high-metabotype-depleted	0.00080433	intermediate
K00672	high-metabotype-associated	0.00081655	intermediate
K00045	high-metabotype-depleted	0.00081947	intermediate
K00619	high-metabotype-depleted	0.00082215	intermediate
K00865	high-metabotype-depleted	0.00082383	intermediate
K01740	high-metabotype-depleted	0.00083694	intermediate
K01736	high-metabotype-depleted	0.00083758	intermediate
K03077	high-metabotype-depleted	0.00084079	intermediate
K01786	high-metabotype-depleted	0.00084079	intermediate
K03080	high-metabotype-associated	0.00084079	intermediate
K01940	high-metabotype-depleted	0.00084371	intermediate
K05349	high-metabotype-depleted	0.0008439	intermediate
K01188	high-metabotype-depleted	0.0008439	intermediate
K05350	Other	0.0008439	intermediate
K02619	high-metabotype-depleted	0.00085224	intermediate
K13020	high-metabotype-depleted	0.00085329	intermediate
K13016	high-metabotype-depleted	0.00085329	intermediate
K09699	Other	0.00085477	intermediate
K00962	high-metabotype-depleted	0.00085601	intermediate

K13954	high-metabotype-depleted	0.00085882	intermediate
K01919	high-metabotype-depleted	0.00085983	intermediate
K11204	Other	0.00085983	intermediate
K03715	high-metabotype-depleted	0.00087051	intermediate
K14333	Other	0.0008716	intermediate
K01232	high-metabotype-depleted	0.00087377	intermediate
K01805	high-metabotype-depleted	0.00087819	intermediate
K02770	high-metabotype-depleted	0.00088325	intermediate
K02769	high-metabotype-depleted	0.00088325	intermediate
K02768	high-metabotype-depleted	0.00088325	intermediate
K01885	high-metabotype-depleted	0.00089055	intermediate
K00769	high-metabotype-depleted	0.00089061	intermediate
K03816	high-metabotype-depleted	0.00089061	intermediate
K06153	high-metabotype-depleted	0.00090729	intermediate
K00122	high-metabotype-depleted	0.00090731	intermediate
K00124	high-metabotype-depleted	0.00090731	intermediate
K00127	high-metabotype-depleted	0.00090731	intermediate
K00123	high-metabotype-depleted	0.00090731	intermediate
K08348	Other	0.00090731	intermediate
K08350	Other	0.00090731	intermediate
K08349	Other	0.00090731	intermediate
K00125	high-metabotype-associated	0.00090731	intermediate
K00126	Other	0.00090731	intermediate
K01658	high-metabotype-depleted	0.00091962	intermediate

K01657	high-metabotype-depleted	0.00091962	intermediate
K14471	Other	0.00093066	intermediate
K14472	Other	0.00093066	intermediate
K00869	high-metabotype-associated	0.00093124	intermediate
K01610	high-metabotype-depleted	0.00093953	intermediate
K01596	high-metabotype-depleted	0.00093953	intermediate
K02302	high-metabotype-depleted	0.00094428	intermediate
K00022	high-metabotype-depleted	0.00094719	intermediate
K00658	high-metabotype-depleted	0.00094926	intermediate
K00258	high-metabotype-associated	0.00094942	intermediate
K00007	Other	0.000952	intermediate
K06445	high-metabotype-depleted	0.00095241	intermediate
K13247	Other	0.00095323	intermediate
K01521	Other	0.00095488	intermediate
K00694	high-metabotype-depleted	0.00095553	intermediate
K00158	Other	0.00095951	intermediate
K03472	high-metabotype-associated	0.00096432	intermediate
K00027	high-metabotype-depleted	0.00096444	intermediate
K00789	high-metabotype-depleted	0.00096808	intermediate
K00625	high-metabotype-depleted	0.0009691	intermediate
K13788	high-metabotype-depleted	0.0009691	intermediate
K02586	high-metabotype-depleted	0.00096999	intermediate
K02591	high-metabotype-depleted	0.00096999	intermediate
K02588	high-metabotype-depleted	0.00096999	intermediate

K01803	high-metabotype-depleted	0.00097036	intermediate
K00302	high-metabotype-depleted	0.00097068	intermediate
K00303	Other	0.00097068	intermediate
K00301	high-metabotype-associated	0.00097068	intermediate
K00314	Other	0.00097068	intermediate
K00886	Other	0.0009731	intermediate
K02535	high-metabotype-depleted	0.00099461	intermediate
K00054	Other	0.00099625	intermediate
K00021	high-metabotype-associated	0.00099625	intermediate
K12112	Other	0.00099674	intermediate
K12111	Other	0.00099674	intermediate
K01694	high-metabotype-depleted	0.0009998	intermediate
K01239	high-metabotype-depleted	0.00100015	intermediate
K01120	high-metabotype-associated	0.00100676	intermediate
K13298	Other	0.00100676	intermediate
K04108	high-metabotype-depleted	0.00101046	intermediate
K01182	high-metabotype-depleted	0.00101251	intermediate
K01089	high-metabotype-depleted	0.0010186	intermediate
K13967	Other	0.00102047	intermediate
K05606	Other	0.00103629	intermediate
K00772	Other	0.00103964	intermediate
K00981	high-metabotype-depleted	0.00103966	intermediate
K00831	high-metabotype-depleted	0.00105027	intermediate
K00319	high-metabotype-associated	0.00105691	intermediate
K12234	high-metabotype-associated	0.00105892	intermediate
K01778	high-metabotype-depleted	0.00107186	intermediate

K01886	high-metabotype-depleted	0.00107586	intermediate
K00526	high-metabotype-depleted	0.00108062	intermediate
K00525	high-metabotype-depleted	0.00108062	intermediate
K00018	high-metabotype-depleted	0.00110198	intermediate
K00015	high-metabotype-depleted	0.00110198	intermediate
K01067	high-metabotype-depleted	0.0011021	intermediate
K00851	high-metabotype-depleted	0.0011091	intermediate
K01012	high-metabotype-depleted	0.00110997	intermediate
K00790	high-metabotype-depleted	0.00111162	intermediate
K00215	high-metabotype-depleted	0.00111185	intermediate
K01431	Other	0.0011166	intermediate
K00006	high-metabotype-depleted	0.00111773	intermediate
K00113	high-metabotype-depleted	0.00111773	intermediate
K00112	high-metabotype-depleted	0.00111773	intermediate
K00111	high-metabotype-depleted	0.00111773	intermediate
K00057	high-metabotype-depleted	0.00111773	intermediate
K03399	Other	0.0011321	intermediate
K03644	high-metabotype-depleted	0.00113491	intermediate
K03801	high-metabotype-depleted	0.00113491	intermediate
K03637	high-metabotype-depleted	0.00113491	intermediate
K03639	high-metabotype-depleted	0.00113491	intermediate
K10815	Other	0.00113526	intermediate
K01034	high-metabotype-depleted	0.0011363	intermediate

K01035	high-metabotype-depleted	0.0011363	intermediate
K01711	high-metabotype-depleted	0.00113631	intermediate
K01790	high-metabotype-depleted	0.00113631	intermediate
K01911	high-metabotype-depleted	0.00113631	intermediate
K03270	high-metabotype-depleted	0.00113631	intermediate
K01904	high-metabotype-depleted	0.00113631	intermediate
K01799	high-metabotype-depleted	0.00113631	intermediate
K12660	high-metabotype-depleted	0.00113631	intermediate
K02301	high-metabotype-depleted	0.00113631	intermediate
K02473	high-metabotype-depleted	0.00113631	intermediate
K07535	Other	0.00113631	intermediate
K00801	Other	0.00113631	intermediate
K03821	Other	0.00113631	intermediate
K01846	high-metabotype-associated	0.00113631	intermediate
K08295	high-metabotype-associated	0.00113631	intermediate
K03894	high-metabotype-associated	0.00113631	intermediate
K06123	high-metabotype-associated	0.00113631	intermediate
K10027	Other	0.00113631	intermediate
K01575	Other	0.00113702	intermediate
K05954	Other	0.00113702	intermediate
K01223	high-metabotype-depleted	0.0011386	intermediate
K01222	high-metabotype-depleted	0.0011386	intermediate
K13017	high-metabotype-depleted	0.00113913	intermediate
K00876	high-metabotype-depleted	0.00114187	intermediate

K00275	high-metabotype-depleted	0.00114741	intermediate
K00868	high-metabotype-depleted	0.00114741	intermediate
K03148	high-metabotype-depleted	0.00115162	intermediate
K01681	high-metabotype-depleted	0.00115396	intermediate
K00884	high-metabotype-associated	0.0011573	intermediate
K00949	high-metabotype-depleted	0.00115737	intermediate
K00457	Other	0.0011621	intermediate
K10217	Other	0.00116651	intermediate
K00208	high-metabotype-depleted	0.00116691	intermediate
K01070	high-metabotype-depleted	0.00117345	intermediate
K05711	Other	0.00118299	intermediate
K06001	high-metabotype-depleted	0.00118907	intermediate
K01695	high-metabotype-depleted	0.00118907	intermediate
K01696	high-metabotype-depleted	0.00118907	intermediate
K07106	high-metabotype-depleted	0.00119113	intermediate
K04110	Other	0.00119243	intermediate
K00692	high-metabotype-associated	0.00119267	intermediate
K05308	Other	0.0011947	intermediate
K01631	Other	0.00121357	intermediate
K01129	high-metabotype-depleted	0.0012137	intermediate
K00765	high-metabotype-depleted	0.00121835	intermediate
K02502	Other	0.00121835	intermediate
K00674	high-metabotype-depleted	0.00121948	intermediate
K03079	high-metabotype-depleted	0.00122248	intermediate
K03082	high-metabotype-depleted	0.00122248	intermediate

K07550	high-metabotype-associated	0.00122665	intermediate
K12252	high-metabotype-associated	0.00122992	intermediate
K00767	high-metabotype-depleted	0.00124839	intermediate
K01807	high-metabotype-depleted	0.00124911	intermediate
K09474	Other	0.00126175	intermediate
K01078	high-metabotype-depleted	0.00126175	intermediate
K01093	Other	0.00126175	intermediate
K03788	Other	0.00126175	intermediate
K03525	high-metabotype-depleted	0.00126694	intermediate
K00867	high-metabotype-depleted	0.00126694	intermediate
K09680	Other	0.00126694	intermediate
K01055	high-metabotype-depleted	0.00128439	intermediate
K00527	high-metabotype-depleted	0.00129247	intermediate
K01721	Other	0.00129537	intermediate
K00631	high-metabotype-depleted	0.00130367	intermediate
K08591	high-metabotype-depleted	0.00130367	intermediate
K03621	high-metabotype-depleted	0.00130367	intermediate
K02227	high-metabotype-depleted	0.00132488	intermediate
K01921	high-metabotype-depleted	0.00132859	intermediate
K01032	Other	0.00133771	intermediate
K01031	high-metabotype-enriched	0.00133771	intermediate
K03527	high-metabotype-depleted	0.00133871	intermediate
K00117	high-metabotype-associated	0.00134421	intermediate
K03186	high-metabotype-depleted	0.00134448	intermediate

K03182	high-metabotype-depleted	0.00134448	intermediate
K01730	high-metabotype-depleted	0.00134775	intermediate
K11180	high-metabotype-depleted	0.00135842	intermediate
K11181	Other	0.00135842	intermediate
K01582	high-metabotype-depleted	0.00136038	intermediate
K02614	high-metabotype-depleted	0.00141688	intermediate
K00048	high-metabotype-depleted	0.00142582	intermediate
K00480	high-metabotype-associated	0.00142638	intermediate
K01612	high-metabotype-depleted	0.00142829	intermediate
K00825	high-metabotype-depleted	0.0014338	intermediate
K00806	high-metabotype-depleted	0.00143799	intermediate
K00539	Other	0.00144136	intermediate
K00848	high-metabotype-depleted	0.0014476	intermediate
K11262	Other	0.00144951	intermediate
K00857	high-metabotype-depleted	0.00145987	intermediate
K12743	Other	0.00146048	intermediate
K01735	high-metabotype-depleted	0.00146744	intermediate
K11541	high-metabotype-depleted	0.00148468	intermediate
K00560	high-metabotype-depleted	0.00151634	intermediate
K00294	high-metabotype-depleted	0.00152415	intermediate
K12304	Other	0.00153481	intermediate
K01190	high-metabotype-depleted	0.00155197	intermediate
K00901	high-metabotype-depleted	0.00155304	intermediate
K01428	high-metabotype-depleted	0.00155489	intermediate

K01429	high-metabotype-depleted	0.00155489	intermediate
K14048	Other	0.00155489	intermediate
K01430	Other	0.00155489	intermediate
K01427	Other	0.00155489	intermediate
K03688	high-metabotype-depleted	0.00156672	intermediate
K00281	high-metabotype-depleted	0.00158514	intermediate
K00282	high-metabotype-depleted	0.00158514	intermediate
K00283	high-metabotype-depleted	0.00158514	intermediate
K00748	high-metabotype-depleted	0.00161117	intermediate
K00794	high-metabotype-depleted	0.00163598	intermediate
K01195	high-metabotype-depleted	0.00165541	intermediate
K01791	high-metabotype-depleted	0.0016575	intermediate
K01092	high-metabotype-depleted	0.00165789	intermediate
K00278	high-metabotype-depleted	0.00165849	intermediate
K03381	high-metabotype-depleted	0.0016606	intermediate
K00788	high-metabotype-depleted	0.00168415	intermediate
K00041	high-metabotype-depleted	0.00169203	intermediate
K00040	Other	0.00169203	intermediate
K03147	high-metabotype-depleted	0.0016946	intermediate
K01961	high-metabotype-depleted	0.00170183	intermediate
K02160	high-metabotype-depleted	0.00170183	intermediate
K01962	high-metabotype-depleted	0.00170183	intermediate
K01963	high-metabotype-depleted	0.00170183	intermediate

K00688	high-metabotype-depleted	0.00170357	intermediate
K00888	Other	0.00170377	intermediate
K00009	high-metabotype-depleted	0.00170553	intermediate
K00131	high-metabotype-depleted	0.00170824	intermediate
K02079	high-metabotype-depleted	0.00170869	intermediate
K01818	high-metabotype-depleted	0.00170975	intermediate
K00939	high-metabotype-depleted	0.00171222	intermediate
K01697	Other	0.00172198	intermediate
K00697	high-metabotype-depleted	0.001728	intermediate
K08687	high-metabotype-associated	0.00173067	intermediate
K08094	high-metabotype-depleted	0.0017331	intermediate
K02509	Other	0.00178265	intermediate
K01439	high-metabotype-depleted	0.00178418	intermediate
K03416	Other	0.00179586	intermediate
K00055	Other	0.00179859	intermediate
K01918	high-metabotype-depleted	0.00180611	intermediate
K08682	high-metabotype-depleted	0.00180857	intermediate
K14154	high-metabotype-associated	0.00181452	intermediate
K00847	high-metabotype-depleted	0.00182242	intermediate
K09251	high-metabotype-depleted	0.00183149	intermediate
K12256	Other	0.00183149	intermediate
K00880	high-metabotype-depleted	0.00183822	intermediate
K02858	high-metabotype-depleted	0.00185841	intermediate
K12409	Other	0.00187821	intermediate
K00190	Other	0.00188076	intermediate

K01839	high-metabotype-depleted	0.0018825	intermediate
K01178	Other	0.00189003	intermediate
K03338	high-metabotype-depleted	0.00190336	intermediate
K01815	high-metabotype-depleted	0.00191432	intermediate
K08302	high-metabotype-depleted	0.00194433	intermediate
K01635	Other	0.00194433	intermediate
K00394	high-metabotype-depleted	0.00195735	intermediate
K00395	Other	0.00195735	intermediate
K00754	high-metabotype-depleted	0.00200128	intermediate
K01218	high-metabotype-depleted	0.00200199	intermediate
K01485	high-metabotype-depleted	0.00200542	intermediate
K00855	high-metabotype-associated	0.00200688	intermediate
K00390	high-metabotype-depleted	0.00202102	intermediate
K01512	high-metabotype-depleted	0.00202319	intermediate
K01270	high-metabotype-depleted	0.00203213	intermediate
K01256	high-metabotype-depleted	0.00203213	intermediate
K01255	high-metabotype-depleted	0.00203213	intermediate
K07751	high-metabotype-associated	0.00203213	intermediate
K11140	Other	0.00203213	intermediate
K01068	Other	0.00206963	intermediate
K12506	high-metabotype-depleted	0.00207224	intermediate
K00677	high-metabotype-depleted	0.00208591	intermediate
K00446	high-metabotype-associated	0.00209512	intermediate
K01194	Other	0.0020994	intermediate
K09023	Other	0.00211616	intermediate

K00517	high-metabotype-associated	0.00211962	intermediate
K01714	high-metabotype-depleted	0.00220089	intermediate
K00164	high-metabotype-depleted	0.00220097	intermediate
K00758	high-metabotype-depleted	0.00220741	intermediate
K00823	Other	0.00222097	intermediate
K03150	high-metabotype-depleted	0.00222557	intermediate
K01621	high-metabotype-depleted	0.00224343	intermediate
K00579	high-metabotype-depleted	0.00225199	intermediate
K00577	Other	0.00225199	intermediate
K00580	Other	0.00225199	intermediate
K00578	high-metabotype-associated	0.00225199	intermediate
K00582	high-metabotype-associated	0.00225199	intermediate
K00581	high-metabotype-associated	0.00225199	intermediate
K00584	high-metabotype-associated	0.00225199	intermediate
K00583	Other	0.00225199	intermediate
K00284	high-metabotype-depleted	0.00226932	intermediate
K00840	high-metabotype-depleted	0.00226982	intermediate
K00673	high-metabotype-depleted	0.00226988	intermediate
K06447	high-metabotype-depleted	0.00227052	intermediate
K01484	Other	0.00227058	intermediate
K03896	high-metabotype-depleted	0.00227122	intermediate
K06920	high-metabotype-depleted	0.00227122	intermediate
K00721	high-metabotype-depleted	0.00227122	intermediate
K01710	high-metabotype-depleted	0.00227122	intermediate

K02291	high-metabotype-depleted	0.00227122	intermediate
K07536	high-metabotype-depleted	0.00227122	intermediate
K00979	high-metabotype-depleted	0.00227122	intermediate
K02549	high-metabotype-depleted	0.00227122	intermediate
K05526	high-metabotype-depleted	0.00227122	intermediate
K01709	high-metabotype-depleted	0.00227122	intermediate
K01772	Other	0.00227122	intermediate
K10011	Other	0.00227193	intermediate
K00436	high-metabotype-depleted	0.00227404	intermediate
K00879	high-metabotype-depleted	0.00227826	intermediate
K14153	high-metabotype-depleted	0.00227991	intermediate
K06162	high-metabotype-depleted	0.00228107	intermediate
K00260	high-metabotype-depleted	0.00229578	intermediate
K00262	high-metabotype-depleted	0.00229578	intermediate
K00261	Other	0.00229578	intermediate
K11540	high-metabotype-depleted	0.00229731	intermediate
K03517	high-metabotype-depleted	0.00230761	intermediate
K05714	Other	0.00232438	intermediate
K01007	high-metabotype-depleted	0.00232532	intermediate
K01006	high-metabotype-depleted	0.00232532	intermediate
K03342	high-metabotype-associated	0.00237473	intermediate
K01808	high-metabotype-depleted	0.00238543	intermediate
K01925	high-metabotype-depleted	0.0023887	intermediate

K01668	high-metabotype-depleted	0.00239651	intermediate
K01690	Other	0.0024007	intermediate
K01637	high-metabotype-associated	0.00241241	intermediate
K01243	high-metabotype-depleted	0.00242435	intermediate
K01920	high-metabotype-depleted	0.00244254	intermediate
K00001	high-metabotype-depleted	0.00244978	intermediate
K13953	high-metabotype-depleted	0.00244978	intermediate
K11754	high-metabotype-depleted	0.00247073	intermediate
K01930	Other	0.00247073	intermediate
K01476	high-metabotype-depleted	0.00247768	intermediate
K00156	high-metabotype-depleted	0.00248673	intermediate
K00177	high-metabotype-depleted	0.00248919	intermediate
K00176	high-metabotype-depleted	0.00248919	intermediate
K00175	high-metabotype-depleted	0.00248919	intermediate
K00174	high-metabotype-depleted	0.00248919	intermediate
K00845	high-metabotype-depleted	0.00252348	intermediate
K00853	high-metabotype-depleted	0.00254621	intermediate
K03272	high-metabotype-depleted	0.00255583	intermediate
K00796	high-metabotype-depleted	0.0025569	intermediate
K13941	high-metabotype-depleted	0.0025569	intermediate
K00766	high-metabotype-depleted	0.00256841	intermediate
K00759	high-metabotype-depleted	0.002577	intermediate
K00912	high-metabotype-depleted	0.00259583	intermediate

K00481	Other	0.00259767	intermediate
K00654	Other	0.00260103	intermediate
K00265	high-metabotype-depleted	0.00260349	intermediate
K00266	high-metabotype-depleted	0.00260349	intermediate
K00264	high-metabotype-depleted	0.00260349	intermediate
K01630	Other	0.0026041	intermediate
K01572	high-metabotype-depleted	0.00260486	intermediate
K01571	high-metabotype-depleted	0.00260486	intermediate
K01573	high-metabotype-depleted	0.00260486	intermediate
K00864	high-metabotype-depleted	0.00260844	intermediate
K01633	high-metabotype-depleted	0.00262567	intermediate
K13940	high-metabotype-depleted	0.00262567	intermediate
K01213	high-metabotype-depleted	0.00262777	intermediate
K00010	high-metabotype-depleted	0.00263066	intermediate
K01647	high-metabotype-depleted	0.00264205	intermediate
K01646	high-metabotype-depleted	0.00264205	intermediate
K01643	high-metabotype-depleted	0.00264205	intermediate
K01644	high-metabotype-depleted	0.00264205	intermediate
K02361	high-metabotype-depleted	0.00264505	intermediate
K02552	Other	0.00264505	intermediate
K00366	high-metabotype-depleted	0.00267454	intermediate
K00363	high-metabotype-depleted	0.00267454	intermediate
K03385	high-metabotype-depleted	0.00267454	intermediate
K00362	Other	0.00267454	intermediate

K11258	high-metabotype-depleted	0.00267856	intermediate
K01653	high-metabotype-depleted	0.00267856	intermediate
K01652	high-metabotype-depleted	0.00267856	intermediate
K01139	Other	0.00268171	intermediate
K00033	high-metabotype-depleted	0.00268686	intermediate
K07514	Other	0.0027072	intermediate
K03331	high-metabotype-enriched	0.00273098	intermediate
K00455	high-metabotype-associated	0.00275136	intermediate
K01686	high-metabotype-depleted	0.0027539	intermediate
K01685	high-metabotype-depleted	0.0027539	intermediate
K01586	high-metabotype-depleted	0.00275435	intermediate
K00690	high-metabotype-depleted	0.00275591	intermediate
K01755	high-metabotype-depleted	0.00275836	intermediate
K01758	high-metabotype-depleted	0.00275934	intermediate
K00680	high-metabotype-depleted	0.00276516	intermediate
K01137	high-metabotype-depleted	0.0027653	intermediate
K00148	Other	0.0027828	intermediate
K13829	high-metabotype-associated	0.0028373	intermediate
K13015	high-metabotype-depleted	0.00284255	intermediate
K01628	high-metabotype-depleted	0.00284606	intermediate
K06163	Other	0.00284958	intermediate
K00797	high-metabotype-depleted	0.00285764	intermediate
K01579	high-metabotype-depleted	0.00286242	intermediate

K00761	high-metabotype-depleted	0.00289069	intermediate
K02825	high-metabotype-depleted	0.00289069	intermediate
K01938	high-metabotype-depleted	0.00289696	intermediate
K01054	high-metabotype-depleted	0.00291339	central
K02203	Other	0.00291491	central
K00702	high-metabotype-depleted	0.00294627	central
K03149	high-metabotype-depleted	0.0029474	central
K01948	high-metabotype-depleted	0.0029544	central
K00926	high-metabotype-depleted	0.0029544	central
K00966	high-metabotype-depleted	0.00296627	central
K00971	high-metabotype-depleted	0.00296627	central
K00384	high-metabotype-depleted	0.00297349	central
K03474	high-metabotype-depleted	0.00298405	central
K01777	high-metabotype-depleted	0.00298415	central
K13830	Other	0.00303206	central
K00036	high-metabotype-depleted	0.00303936	central
K00065	high-metabotype-depleted	0.00304677	central
K05939	high-metabotype-depleted	0.00305725	central
K14578	Other	0.00306217	central
K09473	Other	0.00306353	central
K01555	high-metabotype-depleted	0.00306403	central
K03465	high-metabotype-depleted	0.00309597	central
K01929	high-metabotype-depleted	0.0031022	central

K01895	high-metabotype-depleted	0.00312564	central
K01905	high-metabotype-associated	0.00312564	central
K04781	high-metabotype-associated	0.00312579	central
K00274	high-metabotype-depleted	0.00320178	central
K14152	high-metabotype-associated	0.00321247	central
K03783	high-metabotype-depleted	0.00321895	central
K03784	high-metabotype-depleted	0.00321895	central
K05306	Other	0.00322054	central
K01196	Other	0.00322387	central
K01066	high-metabotype-depleted	0.00326518	central
K01251	high-metabotype-depleted	0.003285	central
K09011	high-metabotype-depleted	0.00333266	central
K02523	high-metabotype-depleted	0.00334012	central
K03524	high-metabotype-depleted	0.00336309	central
K03851	high-metabotype-associated	0.00338408	central
K00230	high-metabotype-depleted	0.00339629	central
K00231	Other	0.00339629	central
K00276	Other	0.00340397	central
K01739	Other	0.00340426	central
K00973	high-metabotype-depleted	0.00340473	central
K01497	high-metabotype-depleted	0.00340473	central
K10026	high-metabotype-depleted	0.00340473	central
K02224	high-metabotype-depleted	0.00340473	central
K08680	high-metabotype-depleted	0.00340473	central

K00978	Other	0.00340473	central
K03897	Other	0.00340473	central
K06167	high-metabotype-depleted	0.00341738	central
K01640	high-metabotype-depleted	0.0034266	central
K01193	high-metabotype-depleted	0.00347429	central
K00822	high-metabotype-depleted	0.00348743	central
K11717	high-metabotype-depleted	0.00350754	central
K00029	high-metabotype-depleted	0.00350801	central
K00028	Other	0.00350801	central
K10218	Other	0.00354819	central
K08660	high-metabotype-depleted	0.00355634	central
K00832	high-metabotype-depleted	0.00356943	central
K00681	high-metabotype-depleted	0.00362737	central
K00795	high-metabotype-depleted	0.00367338	central
K01655	high-metabotype-depleted	0.0036742	central
K01026	high-metabotype-depleted	0.00369603	central
K00764	high-metabotype-depleted	0.00370256	central
K01061	high-metabotype-depleted	0.00374045	central
K01939	high-metabotype-depleted	0.00375783	central
K00492	high-metabotype-associated	0.00378824	central
K00648	high-metabotype-depleted	0.00381001	central
K02437	high-metabotype-depleted	0.00381526	central
K00288	high-metabotype-depleted	0.0038256	central

K01077	high-metabotype-depleted	0.00385906	central
K01113	high-metabotype-depleted	0.00385906	central
K01782	Other	0.00388978	central
K01825	high-metabotype-associated	0.00388978	central
K01812	high-metabotype-depleted	0.00390952	central
K00627	high-metabotype-depleted	0.00392784	central
K12524	high-metabotype-depleted	0.00395109	central
K12525	Other	0.00395109	central
K05774	high-metabotype-depleted	0.00398449	central
K05344	high-metabotype-depleted	0.00399779	central
K01085	Other	0.00399779	central
K01649	high-metabotype-depleted	0.00402178	central
K01426	high-metabotype-depleted	0.00402539	central
K11121	Other	0.00408033	central
K00155	high-metabotype-depleted	0.00409535	central
K01915	high-metabotype-depleted	0.00416016	central
K00605	high-metabotype-depleted	0.00416241	central
K00942	high-metabotype-depleted	0.00417481	central
K13998	Other	0.00417602	central
K01629	high-metabotype-depleted	0.00426003	central
K00940	high-metabotype-depleted	0.00427271	central
K02564	high-metabotype-depleted	0.00429296	central
K00817	high-metabotype-depleted	0.00430096	central
K01738	high-metabotype-depleted	0.00440359	central

K12339	high-metabotype-depleted	0.00440359	central
K01809	high-metabotype-depleted	0.00448319	central
K02495	high-metabotype-depleted	0.00452276	central
K00228	Other	0.00452276	central
K02551	high-metabotype-depleted	0.00453682	central
K01737	high-metabotype-depleted	0.00453682	central
K06042	high-metabotype-depleted	0.00453752	central
K03271	high-metabotype-depleted	0.00453788	central
K02080	high-metabotype-depleted	0.0045537	central
K01058	high-metabotype-depleted	0.00455914	central
K00103	high-metabotype-associated	0.00458637	central
K03519	high-metabotype-depleted	0.00464875	central
K03518	high-metabotype-depleted	0.00464875	central
K03520	high-metabotype-depleted	0.00464875	central
K00198	high-metabotype-depleted	0.00464875	central
K00196	Other	0.00464875	central
K14187	high-metabotype-depleted	0.00467073	central
K00099	high-metabotype-depleted	0.00469363	central
K03384	high-metabotype-associated	0.00474347	central
K00548	high-metabotype-depleted	0.00476349	central
K01787	high-metabotype-depleted	0.00478787	central
K00963	high-metabotype-depleted	0.00480817	central
K01912	high-metabotype-depleted	0.00481492	central

K01744	high-metabotype-depleted	0.00483924	central
K00859	high-metabotype-depleted	0.00494455	central
K10977	high-metabotype-enriched	0.00499419	central
K02777	high-metabotype-depleted	0.00500503	central
K09470	Other	0.00509778	central
K02231	high-metabotype-depleted	0.00510287	central
K14155	high-metabotype-depleted	0.00513218	central
K01760	high-metabotype-depleted	0.00513218	central
K01425	high-metabotype-depleted	0.00518353	central
K02510	high-metabotype-depleted	0.00523287	central
K01114	high-metabotype-depleted	0.00524658	central
K01638	Other	0.005276	central
K12972	Other	0.0052856	central
K00049	Other	0.0052856	central
K13789	high-metabotype-depleted	0.00537469	central
K13787	Other	0.00537469	central
K01495	high-metabotype-depleted	0.00541199	central
K09007	high-metabotype-depleted	0.00541199	central
K00965	high-metabotype-depleted	0.00541754	central
K09698	high-metabotype-depleted	0.00543149	central
K04021	high-metabotype-depleted	0.00550746	central
K05368	high-metabotype-depleted	0.00564712	central
K00299	Other	0.00564712	central
K00595	high-metabotype-depleted	0.00566048	central

K00012	high-metabotype-depleted	0.00566751	central
K00652	high-metabotype-depleted	0.00569649	central
K03183	high-metabotype-depleted	0.00579238	central
K13519	Other	0.00580075	central
K00249	high-metabotype-depleted	0.00581037	central
K14450	Other	0.00584263	central
K00141	Other	0.00592903	central
K03738	high-metabotype-depleted	0.00593055	central
K01595	high-metabotype-depleted	0.00604355	central
K01062	high-metabotype-depleted	0.00607347	central
K00383	high-metabotype-depleted	0.00610751	central
K01752	high-metabotype-depleted	0.00611188	central
K14170	high-metabotype-depleted	0.00614716	central
K07250	high-metabotype-depleted	0.00615098	central
K01081	high-metabotype-depleted	0.0061697	central
K08722	high-metabotype-depleted	0.0061697	central
K03787	high-metabotype-depleted	0.0061697	central
K08723	high-metabotype-depleted	0.0061697	central
K11751	Other	0.0061697	central
K01483	high-metabotype-depleted	0.00624922	central
K03737	high-metabotype-depleted	0.00629242	central
K01501	high-metabotype-depleted	0.00637599	central
K00162	high-metabotype-depleted	0.0063804	central

K00161	high-metabotype-depleted	0.0063804	central
K00163	high-metabotype-depleted	0.0063804	central
K00760	high-metabotype-depleted	0.00643837	central
K00273	Other	0.00646929	central
K00613	high-metabotype-depleted	0.00649727	central
K00821	high-metabotype-depleted	0.00655291	central
K03856	high-metabotype-depleted	0.00675233	central
K01626	high-metabotype-depleted	0.00675233	central
K02318	Other	0.00676442	central
K02233	high-metabotype-depleted	0.00679996	central
K11753	high-metabotype-depleted	0.00682708	central
K00320	high-metabotype-associated	0.0068471	central
K02548	high-metabotype-depleted	0.00691592	central
K00068	high-metabotype-depleted	0.0069177	central
K00132	high-metabotype-depleted	0.00693557	central
K04073	Other	0.00693557	central
K00948	high-metabotype-depleted	0.00704215	central
K03339	Other	0.00704924	central
K01046	high-metabotype-depleted	0.0071999	central
K02563	high-metabotype-depleted	0.00722883	central
K00998	high-metabotype-depleted	0.0072623	central
K00918	Other	0.00731371	central
K01593	high-metabotype-enriched	0.00734986	central
K00946	high-metabotype-depleted	0.00737503	central

K00031	high-metabotype-depleted	0.00737758	central
K00603	high-metabotype-depleted	0.00740237	central
K13821	Other	0.00741901	central
K01756	high-metabotype-depleted	0.00746128	central
K02527	high-metabotype-depleted	0.00759936	central
K01443	high-metabotype-depleted	0.00760708	central
K00850	high-metabotype-depleted	0.00770122	central
K12309	Other	0.00779262	central
K13990	high-metabotype-depleted	0.00779717	central
K13421	Other	0.00780277	central
K01641	Other	0.00787449	central
K01623	high-metabotype-depleted	0.00789328	central
K02226	high-metabotype-depleted	0.00792608	central
K00626	high-metabotype-depleted	0.00792885	central
K01601	high-metabotype-depleted	0.00803579	central
K02372	high-metabotype-depleted	0.00805742	central
K00169	high-metabotype-depleted	0.00812372	central
K00170	high-metabotype-depleted	0.00812372	central
K00172	high-metabotype-depleted	0.00812372	central
K00171	high-metabotype-depleted	0.00812372	central
K01840	high-metabotype-depleted	0.00817984	central
K01719	high-metabotype-depleted	0.00823702	central
K11787	high-metabotype-depleted	0.00823743	central

K00616	high-metabotype-depleted	0.00840804	central
K13497	Other	0.00858134	central
K01624	high-metabotype-depleted	0.00872603	central
K11645	Other	0.00872603	central
K07407	high-metabotype-depleted	0.00878416	central
K07406	high-metabotype-depleted	0.00878416	central
K01189	Other	0.00878416	central
K05597	Other	0.00896878	central
K13831	Other	0.00897011	central
K02517	high-metabotype-depleted	0.00900541	central
K00140	high-metabotype-depleted	0.00900657	central
K05895	Other	0.00905185	central
K00768	high-metabotype-depleted	0.00905255	central
K02428	high-metabotype-depleted	0.00963308	central
K01754	high-metabotype-depleted	0.00974366	central
K00121	high-metabotype-depleted	0.00992738	central
K13038	high-metabotype-depleted	0.00993105	central
K02188	high-metabotype-depleted	0.01017621	central
K04719	Other	0.01017761	central
K12373	high-metabotype-depleted	0.01046929	central
K14157	Other	0.01062354	central
K01000	high-metabotype-depleted	0.01065265	central
K01580	high-metabotype-depleted	0.01079419	central
K03181	Other	0.01082856	central
K13812	high-metabotype-associated	0.010849	central
K14138	high-metabotype-depleted	0.01111507	central

K00814	Other	0.01115534	central
K01599	high-metabotype-depleted	0.01129986	central
K01557	Other	0.0113538	central
K00824	high-metabotype-depleted	0.01136646	central
K07508	Other	0.01180782	central
K13541	high-metabotype-depleted	0.01185361	central
K01053	high-metabotype-associated	0.01199475	central
K00812	high-metabotype-depleted	0.01203587	central
K00811	high-metabotype-depleted	0.01203587	central
K14454	high-metabotype-depleted	0.01203587	central
K00813	high-metabotype-depleted	0.01203587	central
K11358	high-metabotype-depleted	0.01203587	central
K14455	Other	0.01203587	central
K00620	high-metabotype-depleted	0.01206227	central
K00874	high-metabotype-depleted	0.01210147	central
K04042	high-metabotype-depleted	0.01234616	central
K01835	high-metabotype-depleted	0.01240474	central
K00197	high-metabotype-depleted	0.01253785	central
K00194	high-metabotype-depleted	0.01253785	central
K07248	Other	0.01262279	central
K01011	high-metabotype-depleted	0.01294532	central
K00602	high-metabotype-depleted	0.01296137	central
K01492	high-metabotype-depleted	0.01296137	central
K04072	high-metabotype-depleted	0.01319138	central

K03431	high-metabotype-depleted	0.01321643	central
K03179	high-metabotype-depleted	0.01363899	central
K00100	high-metabotype-depleted	0.01404164	central
K05710	Other	0.01408908	central
K00529	high-metabotype-depleted	0.01522539	central
K13540	high-metabotype-depleted	0.01622673	central
K00149	Other	0.016745	central
K00656	high-metabotype-depleted	0.01709293	central
K00129	high-metabotype-depleted	0.01781928	central
K11533	Other	0.01813591	central
K00665	Other	0.01821316	central
K14260	high-metabotype-depleted	0.01847745	central
K00600	high-metabotype-depleted	0.01954724	central
K00632	high-metabotype-depleted	0.02018719	central
K00820	high-metabotype-depleted	0.02096003	central
K01666	high-metabotype-depleted	0.02116003	central
K13799	high-metabotype-associated	0.02174299	central
K00615	high-metabotype-depleted	0.02201963	central
K01622	high-metabotype-depleted	0.02239779	central
K10760	Other	0.02451667	central
K13810	Other	0.02682393	central
K00382	high-metabotype-depleted	0.02709466	central
K13542	high-metabotype-depleted	0.02853149	central
K13853	Other	0.02984132	central
K00830	Other	0.03058833	central

K00002	high-metabotype-depleted	0.03225697	central
K00844	high-metabotype-depleted	0.03388589	central
K01662	high-metabotype-depleted	0.03431382	central
K00128	high-metabotype-depleted	0.03761891	central
K14085	Other	0.03791198	central
K01749	high-metabotype-depleted	0.03893425	central
K01698	high-metabotype-depleted	0.04002135	central
K00643	high-metabotype-associated	0.04195093	central
K01513	high-metabotype-depleted	0.0427719	central
K01625	high-metabotype-depleted	0.04960732	central
K00011	Other	0.05170442	central
K00873	high-metabotype-depleted	0.10409304	central

Table S3. Compound-specific mass spectrometer Multiple reaction monitoring settings

	Retention time (min)	Precursor ion (m/z)	Product ion (m/z)	Product ion (m/z)
**Hydroxycamptothecin-d5**	1.94	587.3	167.2	167.2
**SN-38**	2.141	393	349	293
**SN-38G**	1.738	569	393	349

Table S4. Variance in triplicate injections of SN-38 and SN-38G per sample

Sample	***SN38 (SD) (n = 3)***	***SN38G (SD) (n = 3)***
A	0.003026549	0.829667036
B	0.026210685	0.261309038
C	0.01	1.434428542
D	0.005773503	0.531716318
E	0.002554082	0.887269127
F	0.047604237	0.296118377
G	0.00668431	0.505880147
H	0.025466056	0.003026549
I	0.066109228	0.003026549
J	3.840810763	0.003026549
K	0.289087224	1.48280292
L	0.030989245	0.003026549
M	0.013501111	0.003026549
N	0.033882493	0.003026549
O	0.043321473	0.003026549
P	0.010128343	0.003026549
Q	0.093771638	0.003026549
R	0.084994235	0.003026549
S	*	*
T	2.287552275	0.003026549

* 1 time-point

Supplementary References

1. Wallace, B. D. *et al.* Alleviating cancer drug toxicity by inhibiting a bacterial enzyme. *Science* **330,** 831–5 (2010).
2. Segata, N. *et al.* Metagenomic microbial community profiling using unique clade-specific marker genes. *Nat. Methods* **9,** 811–4 (2012).
3. Parks, D. H., Tyson, G. W., Hugenholtz, P. & Beiko, R. G. STAMP: statistical analysis of taxonomic and functional profiles. *Bioinformatics* **30,** 3123–4 (2014).
4. Feng, Q. *et al.* Gut microbiome development along the colorectal adenoma–carcinoma sequence. *Nat. Commun.* **6,** 6528 (2015).
